# Introduction of Tau Oligomers into Cortical Neurons Alters Action Potential Dynamics and Disrupts Synaptic Transmission and Plasticity

**DOI:** 10.1523/ENEURO.0166-19.2019

**Published:** 2019-10-14

**Authors:** Emily Hill, Thomas K. Karikari, Kevin G. Moffat, Magnus J. E. Richardson, Mark J. Wall

**Affiliations:** 1 School of Life Sciences, University of Warwick, Coventry CV4 7AL, United Kingdom; 2 Institute of Mathematics, University of Warwick, Coventry CV4 7AL, United Kingdom

**Keywords:** hippocampus, long-term potentiation, neocortex, patch clamp, synaptic transmission, tau

## Abstract

Tau is a highly soluble microtubule-associated protein that acts within neurons to modify microtubule stability. However, abnormally phosphorylated tau dissociates from microtubules to form oligomers and fibrils which associate in the somatodendritic compartment. Although tau can form neurofibrillary tangles (NFTs), it is the soluble oligomers that appear to be the toxic species. There is, however, relatively little quantitative information on the concentration-dependent and time-dependent actions of soluble tau oligomers (oTau) on the electrophysiological and synaptic properties of neurons. Here, whole-cell patch clamp recording was used to introduce known concentrations of oligomeric full-length tau-441 into mouse hippocampal CA1 pyramidal and neocortical Layer V thick-tufted pyramidal cells. oTau increased input resistance, reduced action potential amplitude and slowed action potential rise and decay kinetics. oTau injected into presynaptic neurons induced the run-down of unitary EPSPs which was associated with increased short-term depression. In contrast, introduction of oTau into postsynaptic neurons had no effect on basal synaptic transmission, but markedly impaired the induction of long-term potentiation (LTP). Consistent with its effects on synaptic transmission and plasticity, oTau puncta could be observed in the soma, axon and in the distal dendrites of injected neurons.

## Visual Abstract

**Figure:**
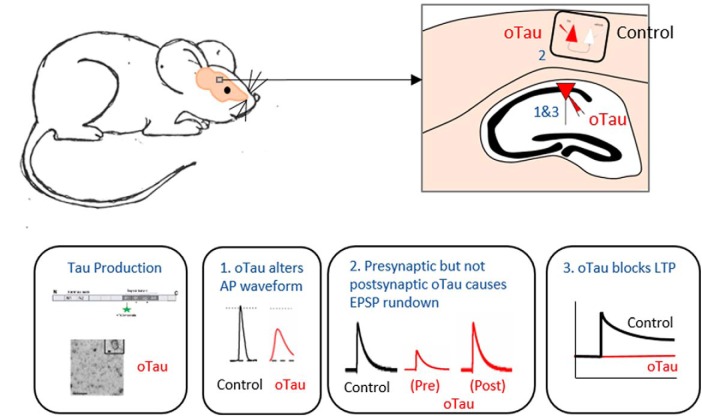

## Significance Statement

The protein tau is highly expressed in neurons and is involved in maintaining neuronal structure. In diseases such as Alzheimer’s disease (AD), tau can form oligomers, which consist of tau molecules joined together. There is growing evidence that these tau oligomers are toxic to neurons, although their precise actions are still being characterized. We have taken the approach of introducing structurally-defined tau-441 oligomers into neurons via the recording electrode (a method previously published by Kaufmann et al., 2016). This method allowed us to provide detailed characterization of the concentration and time-dependent actions of tau oligomers on neuronal properties. We have found that tau interferes with the action potential wave form, modifies synaptic transmission and can block events that probably underlie memory storage.

## Introduction

Tau is a native protein usually associated with microtubules and is key to maintaining cellular morphology, particularly in neuronal axons (Tracy and Gan, 2018). Tau is also expressed in the dendrites and is involved in some forms of synaptic plasticity (Regan et al., 2015). Under physiological conditions, the phosphorylation level of tau protein is regulated by the equilibrated action of kinases and phosphatases. However, dysfunctional states can induce hyperphosphorylation causing tau to disassociate from microtubules, altering their stability (Qiang et al., 2018), with the now free monomeric tau protein prone to aggregation (Avila et al., 2006). The hyperphosphorylated tau monomers initially polymerise to form soluble oligomers. These β-sheet-rich oligomers can then further aggregate into protofibrils, fibrils and neurofibrillary tangles (NFTs). Although NFTs are major histopathological hallmarks of neurodegenerative tauopathies (Nelson et al., 2009), there is strong evidence that the soluble oligomers are the toxic species and play a more important role in disease pathology. Previous studies have shown that tau overexpression leads to neuronal loss, synaptic and behavioral dysfunction without NFT accumulation (Lee et al., 2001; Wittman et al., 2001; Tanemura et al., 2002; Tatebayashi et al., 2002; Andorfer et al., 2003; Spires et al., 2006; Yoshiyama et al., 2007; Cowan et al., 2010). Furthermore, the introduction of tau oligomers (oTau), either extracellularly or via injection into the brain of wildtype rodents induces synaptic, mitochondrial and memory dysfunction (Fá et al., 2016; Ondrejcak et al., 2018), consistent with the hypothesis that oTau are the drivers of toxicity. Alongside the direct effects of oTau, studies have also demonstrated its role in mediating the pathogenesis of other aggregating proteins including amyloid β and α-synuclein (Castillo-Carranza et al., 2015 and Teravskis et al., 2018, respectively).

Many previous electrophysiological investigations of the effect of oTau on neuronal properties have been conducted using extracellular application of oTau or transgenic tauopathy mouse models, where mutant forms of tau protein, that are prone to aggregate, are overexpressed. In such studies, oligomeric tau has been shown to alter the intrinsic excitability of neurons and modulate short-term and long-term plasticity (Tamagnini et al., 2017; Mondragón-Rodríguez et al., 2018; and Hoover et al., 2010; Rocher et al., 2010). For example, Ondrejcak et al. (2018) suggested a postsynaptic action of oTau by showing that administrating intracerebroventricular injections, of either recombinant aggregated tau protein or tau protein isolated from human Alzheimer’s disease (AD) patients, inhibited hippocampal long-term potentiation (LTP) with no effect on paired-pulse ratio. Dissociated tau protein (from viral expression) has also been shown to localize in presynaptic nerve terminals, binding to synaptic vesicles, reducing their mobilization, fusion rate and rate of recycling (Zhou et al., 2017).

In our study, *in vitro* electrophysiology (whole-cell patch clamp recording), together with detailed quantitative analyses has been used to fully characterize the effects of introducing (oTau) directly into cortical neurons. This approach has allowed the evaluation of the direct effects of oTau within a neural network that is free from tau pathology apart from the recorded neuron. Unlike previous studies, this has allowed the delineation of time-dependent and concentration-dependent effects of oTau. Moreover, measuring the electrophysiological and synaptic properties of each neuron immediately after whole-cell breakthrough acts as an internal control for each recording. Using this approach, oTau can be introduced into either presynaptic or postsynaptic cells and the effects on synaptic transmission and plasticity measured. Such targeting is not possible in studies where oTau is applied via the extracellular solution as it is difficult to ascertain whether the observed toxic effects are due to exogenous oTau affecting cell-membrane integrity or the direct intracellular effects of internalized oligomers. While the estimated physiologic concentration of tau protein in neurons is 2 μM (Avila, 2010), here, we demonstrate that introduction of nanomolar concentrations of oTau into hippocampal or neocortical pyramidal neurons is sufficient to cause significant changes in action potential kinetics, impair basal synaptic transmission and disrupt synaptic plasticity over a 45- to 50-min timeframe.

## Materials and Methods

### Protein expression, purification, and characterization

**Figure 1.**
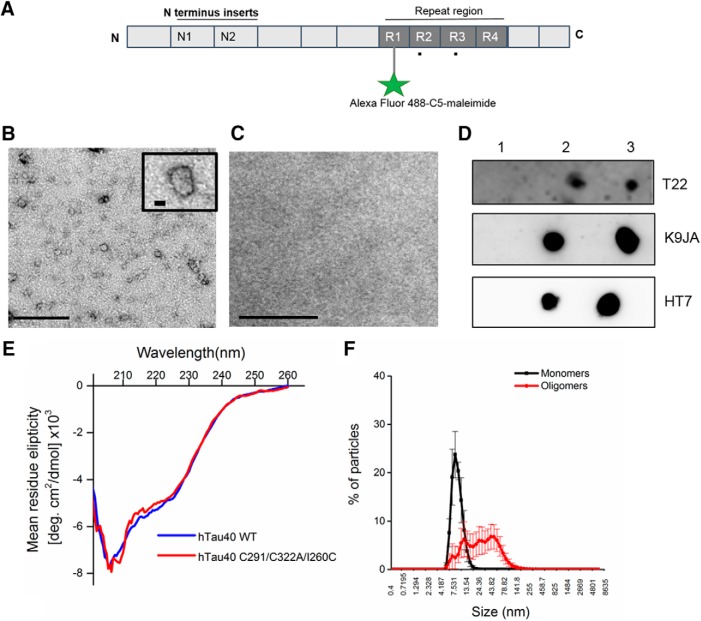
Biochemical characterization of recombinant tau-441oligomers. ***A***, Schematic illustration of full-length tau-441 showing the two N terminus repeats (N1, N2) and the four microtubule-binding repeat domains (R1–R4). To ensure specific labeling with Alexa Fluor 488 C5-maleimide (AF maleimide) on a single cysteine outside the central core of the microtubule repeat region (R1–R4), the two native cysteine residues were each modified to alanine (C291A and C322A, shown as black dots below R2 and R3) and a new cysteine residue introduced at residue 260 (I260C) in R1. This approach has been shown not to have any major effects on the structure and function of tau (see Materials and Methods). ***B***, Representative negative-stain transmission electron microscopy micrograph showing that maleimide-labeled oTau has a granular/spherical conformation. The insert shows a single structure at higher magnification. ***C***, Electron micrograph of negative control (buffer + label only) shows no granular structures. Scale bars = 200 and 20 nm for high-magnification insert. ***D***, Dot blots illustrating the immunoreactivity of oTau that has been dissolved into intracellular recording solution: lane 1, intracellular recording solution alone; lane 2, intracellular recording solution + oTau, which was filtered after the oTau was added; lane 3, intracellular recording solution filtered before addition of oTau. Filtration after addition of oTau decreased the presence of oTau as detected with three different antibodies. Thus, in the electrophysiology experiments, the intracellular solution was filtered before oTau addition. ***E***, CD spectra for oTau showing a prominent negative peak at ∼208 nm, indicating the presence of β sheets. There was no difference observed in spectra with or without the cysteine modifications. ***F***, Dynamic light scattering (DLS) distributions shows that monomeric Tau particles are small with a narrow size distribution (∼80% particles measuring at 7.5–10 nm). In contrast, oTau particles are larger and have a much wider size distribution (∼93% of the particles at 18–32 nm). *Figure Contributions*: Emily Hill and Thomas K. Karikari performed the experiments and analyzed the data.

Briefly, *Escherichia coli* BL21 (DE3) carrying pProEX plasmids (Promega) coding for wild-type full-length tau-441 (Uniprot ID: P10636-8) with N-terminal 6xHis and FLAG tags and cysteine modifications (C291A/C322A/I260C), (Figure 1) were inoculated into Luria broth (15 ml) containing ampicillin (100 μg/ml) and chloramphenicol (35 μg/ml) and incubated at 37°C at 180 rpm overnight. The purpose of the cysteine modifications was to have a single cysteine residue located outside the microtubule-binding region that can be specifically labeled by a fluorophore without potentially interfering with the protein’s functions; this approach has been widely used and shown to have no detrimental effects (Kumar et al., 2014; Michel et al., 2014; Shammas et al., 2015; Karikari et al., 2019). The starter cultures were added to 750-ml fresh LB broth with ampicillin (100 μg ml^−1^) and returned to the shaking incubator for 90 min. When the OD600 reached 0.6, 0. 5mM isopropyl β-D-1 thiogalactopyranoside was added for 1 h. Samples were centrifuged for 10 min at 4°C at 9800 × *g*. The supernatant was removed, and pellets washed with 10 mM sodium phosphate pH 7.4 buffer twice. Pellets were re-dissolved in 10 mM sodium phosphate buffer pH 7.4 and stored at –20°C until use. Samples were purified via immobilized metal affinity chromatography (IMAC).

Eluted fractions from IMAC were analyzed using 6% non-denaturing SDS-PAGE gels to determine the highest yields which were pooled and concentrated using Slide-A-LyzerTM MINI Dialysis devices (10K MWCO; Thermo Scientific). Briefly, the devices were washed with 1-ml sodium phosphate pH 7.4 buffer, the samples added and centrifuged for 30 min at 2600 × *g* at 4°C. A bicinchoninic acid assay (#786-570, G-Biosciences) was used to calculate the concentration and where needed the remaining liquid concentrated with further spins.

### Preparation of fluorescently labeled oTau

Purified Tau-441 was treated with 5x molar excess of tris(2-carboxyethyl)phosphine (TCEP) for 1 h, and then with 4× molar excess of Alexa Fluor-maleimide (#A10254, Invitrogen) overnight in the presence of sodium phosphate buffer pH 7.4. Free fluorophore and reducing agent were removed by 5 × 2 h repeat dialysis against 2 l of dialysis buffer (50 mM Tris HCl, pH 7.5, and 100 mM NaCl) in a Slide-A-LyzerTM MINI Dialysis device (10K MWCO) at each step. Non-denaturing SDS-PAGE followed by ultraviolet light exposure were used to confirm labeling, with efficiency spectrophotometrically estimated using Beer’s law and molar extinction coefficient of tau-441. Unlabeled controls were prepared following the same protocol but with equal volume of 10 mM sodium phosphate buffer pH 7.4 instead of the maleimide label. The entire labeling process was performed at room temperature.

### Circular dichroism (CD) spectroscopy

CD spectra were collected on unlabeled tau-441 diluted to 10 μM in sodium phosphate buffer pH 7.4. The sample was loaded in a 1-mm path-length cell and transferred to a Jasco J-815 CD spectropolarimeter. Ten different spectra were taken on each sample and the average presented. The analytical conditions were: scan speed 100 nm/min, response time 1 s, data pitch 0.1 nm, and high-tension voltage ≤550 V.

### Transmission electron microscopy

Formvar/carbon-coated 300-mesh copper grids (#S162, Agar Scientific) were glow-discharged using the ELMO system from Cordouan Technologies. Five microliters of labeled or unlabeled tau-441 preparations were pipetted onto the grid and allowed to bind for 1 min. Excess samples were removed with a strip of filter paper, and 5 μl of 2% uranyl acetate added for 1 min. After removing the excess stain with a strip of filter paper, the grids were imaged using a JEOL-2100F transmission electron microscope.

### Dynamic light scattering

Size distributions of labeled proteins at 1 mg/ml were measured on a Zetasizer Nano ZS machine (Malvern). Up to ten repeat measurements were obtained for each sample. The “number distribution” function was used to compute the percentage size distribution of the particles.

### Dot blot

Two microliter aliquots of tau-441 (444 μM) dissolved in intracellular patching solution were spotted onto nitrocellulose membranes, allowed to dry and then blocked with 10% non-fat milk in PBS with 0.05% Tween for 1 h. The membranes were thereafter washed five times with 10% Tris-buffered saline (TBS)-Tween and incubated for 2 h with the primary antibodies diluted in PBS-Tween: T22 (#ABN454, Merck; 1:1000 dilution), HT7 (#MN1000, ThermoFisher; 1:1000 dilution), K9JA (#A0024, Dako; 1:5000 dilution). Subsequently, the membranes were re-washed five times with 10% TBS-Tween and then treated for 2 h with secondary antibody (anti-rabbit IgG #31450 or anti-mouse #62-6520, ThermoFisher). Following further washes, the membranes were developed with an electrochemiluminiscent detection kit (BIORAD Clarity Western ECL #170-5060) and imaged.

### Preparation of tau monomers

oTau was incubated with 5 mM DTT (Sigma-Aldrich) for 30 min at 60°C to induce the breakdown to monomers (Fá et al., 2016). This conformational change was confirmed by non-denaturing SDS-PAGE w 6% gels with protein bands detected after staining with Instant Blue (Expedeon). All whole-cell patch experiments involving the introduction of monomeric tau were completed within 3 h of the monomerization protocol. To confirm that the tau injected into neurons was monomeric, aliquots from the same monomerization procedure were mixed with the intracellular patch solution and resolved on SDS-PAGE gels as described above.

### Electrophysiology

#### Preparation of hippocampal and neocortical brain slices

All experiments were approved by the local Animals Welfare and Ethics Board (AWERB) at the University of Warwick. Male BL6 mice (approximately three to four weeks and P12–P21 for paired-synaptic transmission studies) were killed by cervical dislocation and decapitated in accordance with the United Kingdom Animals (Scientific Procedures) Act (1986). Parasagittal hippocampal and neocortical slices (350 μM) were cut with a Microm HM 650V microslicer in cold (2–4°C) high Mg^2+^, low Ca^2+^ aCSF, composed of the following: 127 mM NaCl, 1.9 mM KCl, 8 mM MgCl_2_, 0.5 mM CaCl_2_, 1.2 mM KH_2_PO_4_, 26 mM NaHCO_3_, and 10 mM D-glucose (pH 7.4 when bubbled with 95% O_2_ and 5% CO_2_, 300 mOsm). Neocortical slices were cut at an angle of +15°, such that the blade started cutting from the surface (layer 1) of the neocortex toward the caudal border of the neocortex [to ensure the integrity of Layer V pyramidal cell (Layer V PC) dendrites; Kerr et al., 2013]. Slices were stored at 34°C in standard aCSF (1 mM Mg^2+^ and 2 mM Ca^2+^) for between 1 and 8 h.

#### Whole-cell patch clamp recording from pyramidal cells

A slice was transferred to the recording chamber, submerged and perfused (2–3 ml/min^−1^) with aCSF at 30°C. Slices were visualized using IR-DIC optics with an Olympus BX151W microscope (Scientifica) and a CCD camera (Hitachi). Whole-cell current-clamp recordings were made from pyramidal cells in area CA1 of the hippocampus and from thick- tufted Layer V PCs in the somatosensory cortex using patch pipettes (5–10 MΩ) manufactured from thick walled glass (Harvard Apparatus). Pyramidal cells were identified by their position in the slice, morphology (from fluorescence imaging) and characteristics of the standard current−voltage relationship. Voltage recordings were made using an Axon Multiclamp 700B amplifier (Molecular Devices) and digitized at 20 kHz. Data acquisition and analysis were performed using pClamp 10 (Molecular Devices). Recordings from neurons that had a resting membrane potential of between –60 and –75 mV at whole-cell breakthrough were accepted for analysis. The bridge balance was monitored throughout the experiments and any recordings where it changed by >20% were discarded. Tau-protein oligomers, from a 22 μM stock (monomer concentration), were added to filtered intracellular solution containing the following: 135 mM potassium gluconate, 7 mM NaCl, 10 mM HEPES, 0.5 mM EGTA, 10 mM phosphocreatine, 2 mM MgATP, and 0.3 mM NaGTP (293 mOsm, pH 7.2) to give a final concentration of either 44, 133, or 444 nM tau-protein oligomers for CA1 neurons (2, 6, and 20 μg/ml tau) and 666 nM for Layer V PCs (30 μg/ml tau). Intracellular solution was filtered before the addition of tau-protein oligomers.

### Stimulation protocols

To extract the electrophysiological properties of recorded neurons, both step and more naturalistic, fluctuating currents were injected at 10-min intervals for a duration of the recordings as in (Kaufmann et al., 2016).

#### Standard IV protocol

**Figure 2.**
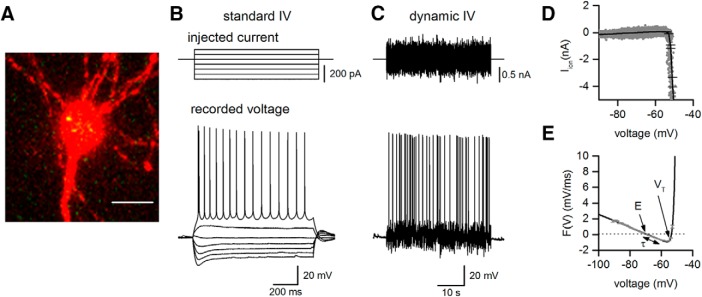
oTau localization in injected neurons and extraction of electrophysiological parameters. ***A***, Micrograph of a labeled hippocampal CA1 pyramidal cell (red) with oTau (green) present in the cell body. The tau protein is labeled with Alexa Fluor 488 maleimide and the neuron is filled with Alexa Fluor 594 dye. Scale bar = 14 μm. ***B***, Illustration of the standard I-V protocol used to extract neural parameters: current steps start from –200 to –300 pA and are increased by 50 pA (top panel) until a regular firing pattern is induced (bottom panel). Current steps around the resting potential were used to extract the input resistance. ***C***, The dynamic I-V protocol injects a naturalistic current into the cell (top panel) and the voltage recorded (bottom panel) is used to extract a set of parameters using the dynamic I-V method and to determine the firing rate. ***D***, Mean ionic current I_ion_ is plotted against membrane potential (gray). The black line is the dynamic I-V curve generated by the average current at a particular voltage (in 1-mV bins). ***E***, The negative of I_ion_/C is then plotted (gray) along with the EIF computational model fit, black line. From this curve a number of subthreshold parameters can be extracted (such as resting potential E, time constant τ, and spike-threshold voltage V_T_). *Figure Contributions*: Emily Hill performed the experiments and analyzed the data.

The standard current−voltage relationship was constructed by injecting step currents from –200 pA (CA1 pyramidal cells) and –600 to –400 pA (Layer V PCs) incrementing by either 50 or 100 pA until a regular firing pattern was induced (Fig. 2). A plot of step current against voltage response around the resting potential was used to measure the input resistance (gradient of the fitted line).

#### Dynamic I-V protocol

The dynamic-I-V curve, defined by the average transmembrane current as a function of voltage during naturalistic activity can be used to efficiently parameterize neurons and generate reduced neural models that accurately mimic the cellular response. The method has been previously described (Badel et al., 2008; Harrison et al., 2015; Kaufmann et al., 2016); for the dynamic-IV computer code, see Harrison et al. (2015). Briefly, a current wave form, designed to provoke naturalistic fluctuating voltages, was constructed using the summed numerical output of two Ornstein–Uhlenbeck processes (Uhlenbeck and Ornstein, 1930) with time constants τ_fast_ = 3 ms and τ_slow_ = 10 ms. This current wave form, which mimics the stochastic actions of AMPA and GABA-receptor channel activation, is injected into cells and the resulting voltage recorded (a fluctuating, naturalistic trace). The voltage trace was used to measure the frequency of action potential firing and to construct a dynamic-I-V curve. The firing rate was measured from voltage traces evoked by injecting a current wave form of the same gain for all recordings (firing rate ∼2–3 Hz). Action potentials were detected by a manually set threshold and the interval between action potentials measured. Dynamic I-V curves were constructed and used to extract a number of parameters including the capacitance, time constant, input resistance, resting membrane potential, spike threshold and spike onset (Fig. 2; Badel et al., 2008; Kaufmann et al., 2016). Using these parameters in a refractory exponential integrate-and-fire (rEIF) model reliably mimics the experimental data, with a spike prediction of ∼70–80% as shown previously (Badel et al., 2008). All analyses of the dynamic-I-V traces were completed using either MATLAB or Julia software platforms (Bezanson et al., 2017).

### Synaptic transmission

To measure synaptic transmission between connected neighboring neocortical thick-tufted Layer V PCs, two to three simultaneous whole-cell current-clamp recordings were made in somatosensory cortex (Markram et al., 1997; Kerr et al., 2013). Recordings were made from neurons in slices from P12 to P21 mice because unitary EPSPs have a larger amplitude than unitary EPSPs in slices from older mice and show marked short-term depression (Reyes and Sakmann, 1999; Kerr et al., 2013) which can be used to measure the effects of oTau on release probability. Once synaptic connectivity was detected, six action potentials were evoked in the presynaptic neuron (5 at 20 Hz followed by a single recovery action potential after a 1 s interval) using 5 ms current steps. These stimulus trains were separated by 10 s and repeated for the duration of recordings. The amplitude of overlapping unitary EPSPs was accurately measured using voltage deconvolution and reconvolution (Richardson and Silberberg, 2008; Kerr et al., 2013).

To measure LTP, whole-cell current-clamp recordings were made from CA1 hippocampal pyramidal cells in the presence of 50 μM picrotoxin (to block GABA_A_ receptors). Schaffer collaterals were stimulated with a concentric bipolar electrode (FHC) every 20 s and after a 15 min baseline, LTP was induced by θ-burst stimulation [TBS; 10 trains of 10 stimuli (100 Hz) separated by 100 ms]. The stimulation strength was set to evoke reliable and robust synaptic transmission (EPSP amplitude ∼3 mV) without producing action potential firing in the postsynaptic cell.

### Statistical analysis

Analysis was performed using non-parametric Wilcoxon rank-sum tests and ANOVAs in GraphPad Prism. In the text, values are given as mean ± SEM. Mean ± SD are given in Table 1 and Extended Data Table 1-1.

**Table 1.** Electrophysiological parameters measured for CA1 hippocampal pyramidal cells at time 0 for all experimental treatments

	Vehicle			BSA			44 nM oTau			133 nM oTau			444 nM oTau		
Parameter	Mean	SEM	SD	Mean	SEM	SD	Mean	SEM	SD	Mean	SEM	SD	Mean	SEM	SD
C (pF)	121.8	± 12.56	± 62.8	125.6	± 14.17	± 34.43	132.55	± 17.65	± 79.43	105	± 8.04	± 44.22	125.3	± 14.31	± 71.55
R in (MΩ)	164.4	± 12.22	± 61.1	183.6	± 21.13	± 52.83	157.89	± 23.01	± 103.55	180.36	± 20.42	± 112.31	176.8	± 4.74	± 23.7
τ (ms)	20.72	± 1.75	± 8.75	15.72	± 0.89	± 2.23	16.67	± 1.86	± 8.37	15.24	± 1.40	± 7.7	19.63	± 2.25	± 1.25
E (mV)	–67.2	± 1.16	± 5.8	–68.5	± 1.34	± 3.35	–69.63	± 0.91	± 4.10	–66.22	± 1.06	± 5.83	–64.95	± 2.0	± 10
V T (mV)	–50.07	± 0.68	± 3.4	–52.69	± 1.26	± 3.15	–54.8	± 0.984	± 4.43	–50.67	± 1.35	± 7.43	–50.92	± 1.49	± 7.45
ΔT (mV)	0.77	± 0.036	± 0.18	0.82	± 0.063	± 0.16	0.97	± 0.15	± 0.68	0.87	± 0.1	± 0.55	0.89	± 0.08	± 0.4
Action potential Amplitude (mV)	75.8	± 4.87	± 24.35	83.6	± 1.84	± 4.6	86.11	± 1.59	± 7.16	77.91	± 2.82	± 15.51	82.66	± 1.9	± 9.5
Duration (ms)	1.42	± 0.093	± 0.465	1.48	± 0.07	± 0.18	1.28	± 1.31	± 5.90	1.41	± 0.07	± 0.385	1.44	± 0.053	± 0.265
Rise (mV/ms)	249.4	± 30.54	± 152.7	288.4	± 17.8	± 44.5	318.8	± 26.55	± 119.48	252.7	± 23.68	± 130.24	282.11	± 21.85	± 109.25

See Extended Data Table 1-1 for the mean, SEM, and SD for all other recorded parameters.

10.1523/ENEURO.0166-19.2019.t1-1 Extended Data Table 1-1 Electrophysiological parameters measured for single layer V cells, during paired layer V recordings and for CA1 neurons in the long-term potentiation experiments. Data is shown as mean, standard error of the mean (SEM) and standard deviation (SD). Download Table 1-1, DOC file.

### Immunohistochemistry, localization of tau protein

Alexa Flour 594 hydrazide dye (Invitrogen) was added to the intracellular solution (0.05 mM final concentration) to allow cell visualization. CA1 pyramidal neurons injected with labeled oTau were recorded for a minimum of 20 min to allow the diffusion of tau protein out of the pipette and into the cell. The pipette was then carefully removed from the cell, slices were fixed in paraformaldehyde (PFA; 4%) overnight at 4°C and washed five times for 5 min in PBS the next morning. Slices were mounted and fixed using Vectashield (Vector Labs). A Leica 710 confocal microscope was used for imaging and Zen software for image processing. A subset of 4 neurons were fully reconstructed using the Airyscan module (tiled *z*-stacks consisting of ∼28 stacks each of 260 *z*-planes) using a Leica 880 confocal microscope to investigate the distribution of oTau in the axon and dendrites.

### Drugs

Picrotoxin (Sigma) and trans-2-carboxy-5,7-dichloro-4-phenylaminocarbonylamino-1,2,3,4-tetrahydroquinoline (L689,560; Hello-Bio) were made as stock solutions (1–50 mM) and diluted in aCSF on the day of use.

## Results

### Production and biochemical characterization of human oTau

Recombinant full-length human tau-441, the longest tau isoform expressed in the human central nervous system (Fig. 1*A*), was expressed in *E. coli*, labeled, oligomerized, and characterized (as demonstrated in Karikari et al., 2017, 2019). The tau preparation was labeled with Alexa Fluor 488-C5-maleimide to block the formation of high molecular-weight aggregates and fibrils and to allow the tracking of oTau in recorded neurons. The expressed oTau had a spherical/granular structure, as shown by negative-stain electron microscopy (Fig. 1*B*,*C*). CD spectroscopy showed a single negative peak at ∼208 nm (Fig. 1*E*), an intermediate between those peaks typical for monomers and filaments (200 and 220 nm, respectively), suggesting the presence of β-sheet structures (von Bergen et al., 2005, Karikari et al., 2017). The CD spectrum was similar when the cysteine residues were not mutated showing that this alteration had little effect on secondary structure (Fig. 1*E*). Dynamic light scattering showed that monomeric Tau had a uniform small size (mean size = 8 ± 3.2 nm, with ∼80% of the particles having a size between 7.5 and 10 nm). In contrast, oTau was larger and had a wider size distribution with ∼93% particles measuring between 18 and 32 nm (Fig. 1*F*). oTau that was diluted into intracellular recording solution reacted strongly with the T22 oligomer-preferring antibody (Fig. 1*D*), thus the tau protein remains in an oligomeric form. The same oTau preparations also reacted strongly with HT7 and K9JA antibodies, which bind to the mid-region and microtubule-binding region tau, respectively (Fig. 1*D*).

### Effects of oligomeric tau on CA1 pyramidal cell subthreshold electrophysiological properties

Two controls were used in the electrophysiological recording experiments. First, a vehicle solution (sodium phosphate pH 7.4 buffer), treated with the same labeling protocol as tau protein, was added to the intracellular solution (at the same volume as the oTau). Second, to ensure that the addition of a protein to the intracellular solution did not produce non-specific effects, bovine serum albumin (BSA) was added to the intracellular solution (20 μM). BSA has a similar molecular-weight to tau monomers and was injected at higher concentration than oTau (20 μM vs 44–666 nM).

**Figure 3.**
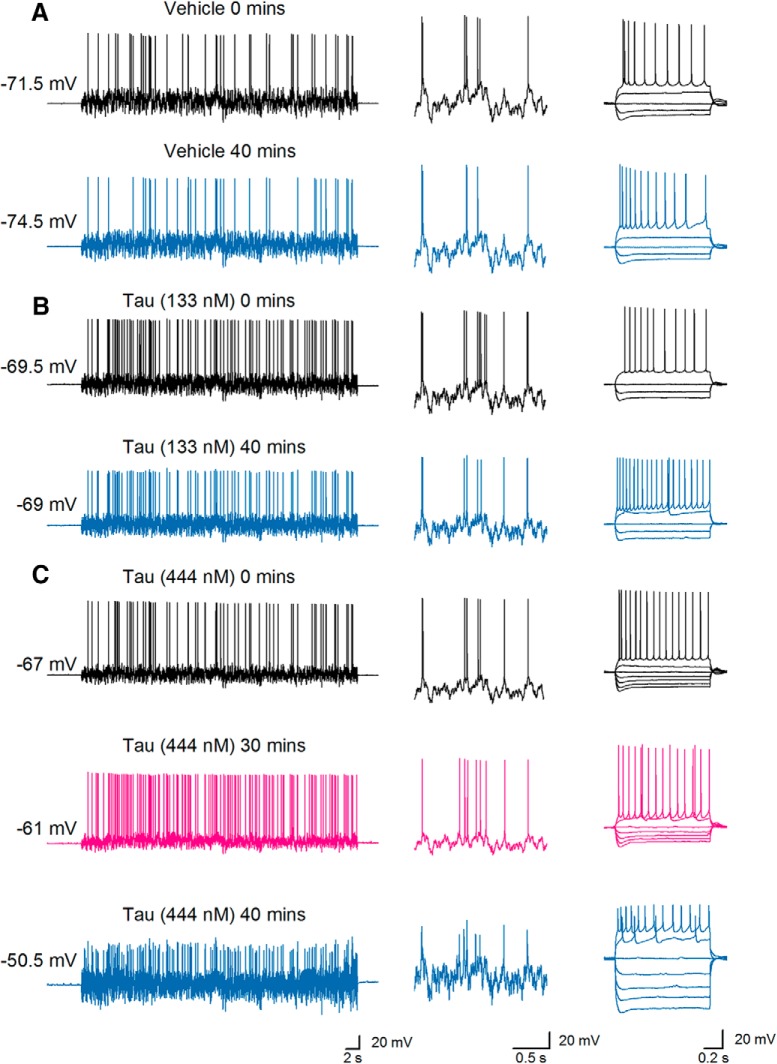
oTau induces little change in sub-threshold electrophysiological parameters until late time points. ***A***, The membrane-potential response to naturalistic current injection (left panel) from a hippocampal CA1 pyramidal cell injected with vehicle at time 0 (after whole-cell breakthrough, top) and after 40 min of recording (bottom). The inset shows parts of the membrane-potential response at an expanded time base illustrating that there is no significant change in the voltage response to naturalistic current injection over time. The standard current−voltage response (right panel) also does not change during recording. ***B***, The membrane-potential response to naturalistic current injection (left panel) from a pyramidal cell injected with oTau (133 nM) at time 0 (after whole-cell breakthrough, top) and after 40 min of recording (bottom). The inset shows parts of the membrane response at an expanded time base illustrating little change in the voltage response to naturalistic current over time although the action potential amplitude is smaller. Right panel, The standard current−voltage responses at time 0 and after 40 min. ***C***, The membrane-potential response to naturalistic current injection (left panel) from a pyramidal cell injected with oTau (444 nM) at time 0 (after whole-cell breakthrough), after 30 and 40 min of recording. There was a depolarization over the time period leading to an increased firing rate at 30 and 40 min. The inset shows parts of the voltage response at an expanded time base illustrating little change in the electrophysiological properties at 30 min but clear changes at 40 min. Right panel, The standard current−voltage response at time 0 and after 30 and 40 min. There are clear changes in the standard current−voltage response (a marked increase in input resistance) after 40 min of recording (right panels) but not after 30 min. *Figure Contributions*: Emily Hill performed the experiments and analyzed the data.

The two controls and oTau, each diluted in the intracellular solution, were introduced into hippocampal CA1 pyramidal cells via the recording electrode during whole-cell current-clamp recording. Following the termination of recordings (after ∼50 min), slices were fixed and the recorded cells visualized with confocal microscopy. The presence of oTau could be observed in the somatic compartment of recorded neurons (Fig. 2*A*). Standard and dynamic IV protocols (Fig. 2*B*,*C*) were used to extract electrophysiological parameters from neurons where different concentrations of oTau (44, 133, and 444 nM), vehicle or BSA had been introduced. Electrophysiological parameters were then compared between the first time point (0–5 min after whole-cell breakthrough) and at 40 min, where the majority of recordings were still stable. At the initial time point, Kruskal–Wallis tests confirmed that there were no significant differences in any of the electrophysiological parameters (resting potential, input resistance, capacitance, time constant, spike threshold, and spike onset) from neurons injected with the different agents (*n* = 10 slices for vehicle, *n* = 5 slices for BSA, *n* = 9 slices for oTau 44 nM, *n* = 11 slices for oTau 133 nM, and *n* = 10 slices for oTau 444 nM; Table 1). Therefore, the initial quality of recordings and neural properties were comparable across all the experimental treatments. Most of the extracted parameters did not significantly change over the duration of recordings for cells that had either been injected with vehicle, BSA, 44 nM, or 133 nM oTau (Fig. 3*A*,*B*). However, there was a significant increase in input resistance (176.8 ± 4.73 to 239.3 ± 36.3 mΩ, *p* = 0.0020) and depolarization of the resting potential (–67.98 ± 1.69 mV at 0 min and –61.79 ± 1.655 mV, *p* = 0.0494) measured after 40 min for neurons injected with 444 nM oTau (Fig. 3*C*). Consistent with this increase in input resistance and membrane potential depolarization, there was also a significant increase in the action potential firing rate in cells injected with 444 nM oTau (*p* = 0.0254), and although not reaching overall significance, six out of seven cells injected with 133 nM oTau also had an increased firing rate.

### oTau markedly slows action potential dynamics and reduces action potential amplitude

**Figure 4.**
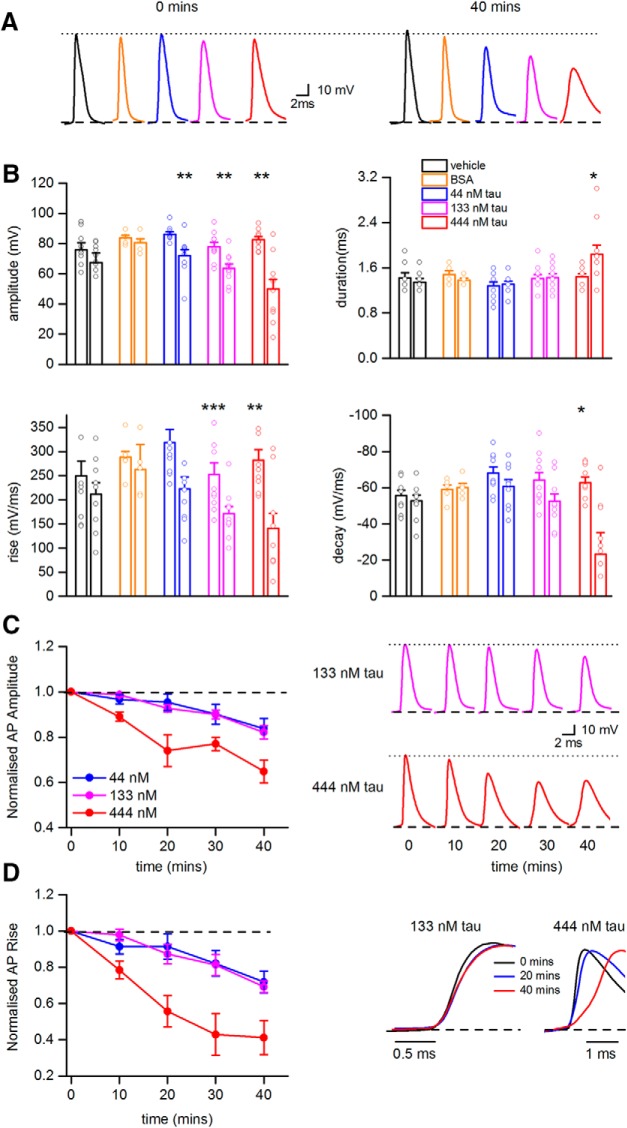
oTau significantly changes action potential dynamics. ***A***, Examples of action potential waveforms recorded at time 0 (left panel) and after 40 min (right panel) from hippocampal CA1 pyramidal cells injected with either vehicle, BSA, or oTau (44, 133, or 444 nM). ***B***, Summaries of the changes in action potential parameters (amplitude, duration, rate of rise, and rate of decay) from recordings with neurons injected with either vehicle, BSA, or oTau (44, 133, or 444 nM). For each treatment there are two bars with associated data points (mean values from single recordings). The first bar and associated points are from time 0 and the second bar and associated points are after 40 min of recording. Amplitude was significantly decreased in all three tested concentrations of tau between time points 0 and 40 min (44 nM *p* = 0.0039, 133 nM *p* = 0.0020, 444 nM *p* = 0.0028). Action potential width increased significantly in cells where 444 nM oTau was introduced (*p* = 0.0156). The rate of rise and decay were also altered following introduction of oTau. Rise was significantly slower for all three concentrations of oTau (44 nM *p* = 0.0641, 133 nM *p* = 0.00054, and 444 nM *p* = 0.002228). However, decay was significantly slower only for 444 nM oTau, *p* = 0.012. ***C***, Left panel shows the relative change in action potential amplitude plotted against time for cells injected with oTau (44, 133, or 444 nM). Action potential amplitudes are normalized to the amplitude at time 0. Right panel shows examples of average action potential waveforms recorded at different time points from neurons injected with either 133 or 444 nM oTau illustrating the change in amplitude. ***D***, Left panel shows the relative change in action potential rise against time for neurons injected with oTau (44, 133, or 444 nM). The speed of rise has been normalized to the speed of rise of action potentials recorded at time 0. Right panel shows superimposed and normalized action potential waveforms illustrating the effects of oTau at 133 and 444 nM on the rate of action potential rise against recording time. *P < 0.05, **P < 0.01, ***P < 0.001.*Figure Contributions:* Emily Hill performed the experiments and analyzed the data.

The dynamic IV parameter extraction was effective for all conditions at 0 min (mean spike match of 72.2 ± 1.7%, predicted vs experimental data). However, for the cells with oTau introduced, by 40 min, the model predictions were unable to accurately match the experimental spike data (mean spike match of 46.1 ± 5.5%) and therefore was not used to extract parameters at later time points. To establish why there might have been a fall in spike match efficiency, we looked at whether there were changes to the parameters that define the action potential wave form (amplitude, duration, rate of rise, and decay). There was no significant difference in any of these parameters at time 0 across all of the experimental treatments using Kruskal–Wallis tests (Table 1). However significant changes in the action potential wave form were observed at the 40 min timepoint for all of the concentrations of oTau (Fig. 4*A*,*B*) but were not observed when neurons were injected with either vehicle or BSA (Fig. 4*A*,*B*). For all three concentrations of oTau (44, 133, and 444 nM), there was a significant decrease in action potential amplitude, speed of rise and speed of decay at 40 min compared to time 0 (Fig. 4*B*). The time course for these changes in action potential parameters (amplitude and rate of rise) were examined for the different concentrations of oTau (Fig. 4*C*,*D*). The effects of 444 nM oTau occurred significantly earlier than for 44 and 133 nM oTau (10 vs 20 min), characteristic of concentration-dependent effects.

### The changes in action potential amplitude and kinetics do not occur with monomeric tau protein

**Figure 5.**
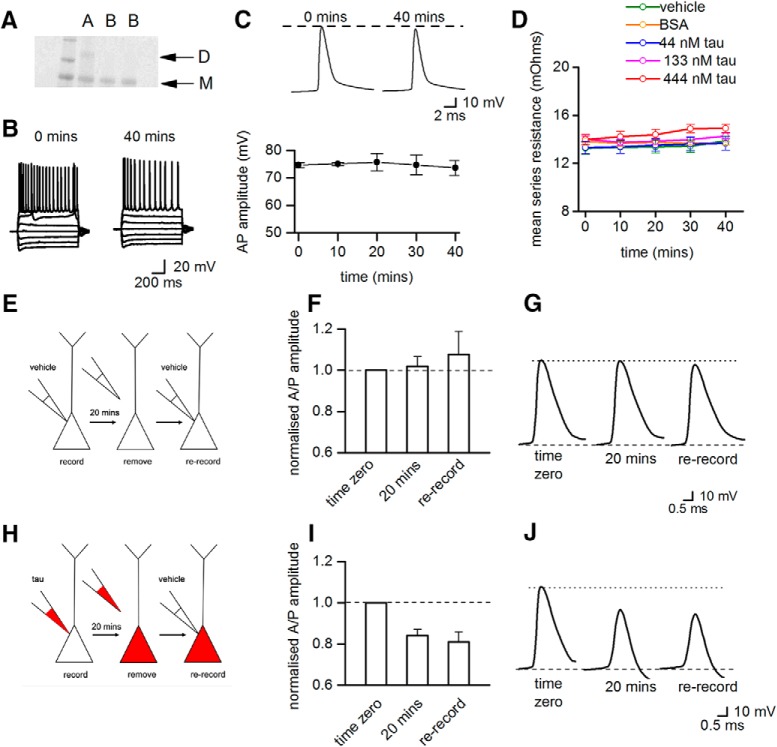
Monomeric tau protein does not change action potential dynamics and the accumulation of oTau in the pipette cannot account for the effects on action potential dynamics. ***A***, Gel showing dimeric tau protein (lane A) and tau protein converted to monomers (mTau, in lanes labeled B). ***B***, There is no significant difference between current−voltage responses from a hippocampal CA1 neurons injected with 444 nM mTau measured at time 0 and after 40 min of recording. ***C***, Top panel shows examples of action potential waveforms recorded at time 0 and after 40 min of recording from a neuron injected with 444 nM mTau. The mTau does not change the amplitude or kinetics of the action potential waveform. ***C***, Bottom panel summarizes data from five neurons, showing that action potential amplitude does not change over time when neurons are injected with 444 nM mTau. ***D***, The mean series resistance plotted against time for neurons injected with vehicle, BSA, or oTau (44, 133, or 444 nM). There was no significant difference in series resistance between treatments and the series resistance did not significantly increase during recordings. ***E***, Diagram illustrating the control re-patching protocol. Pyramidal-cell recordings were made (20 min) with vehicle present in the intracellular solution. The patch-electrode was carefully removed from the cell and then the cell was re-recorded from using a new patch-electrode also containing vehicle. ***F***, There was no change in action potential amplitude during the initial recording (at 20 min, action potential amplitude was 102 ± 4% of the amplitude at time 0) and after re-recording with a new electrode (108 ± 11% of amplitude at time 0, *n* = 4 neurons). Thus, the re-patching protocol itself does not induce changes in action potential amplitude. ***G***, Examples of average action potential waveforms from a single recording. ***H***, Diagram illustrating the test re-patching protocol. Pyramidal cell recordings were made (20 min) with oTau (444 nM) in the intracellular solution. The patch-electrode was then carefully removed from the cell and the cell was then re-recorded from using a patch-electrode containing intracellular solution with vehicle. ***I***, In cells injected with oTau (444 nM), action potential amplitude decreased during the initial recording period (at 20 min, action potential amplitude was 84 ± 3.1% of the amplitude at time 0) and remained depressed following re-recording with a new patch-electrode containing vehicle (81 ± 5% of amplitude at time 0, *n* = 3). ***J***, Shows examples of average action potential waveforms from a single re-patching experiment with 444 nM oTau. These control experiments strongly suggest that the aggregation of oTau in the electrode or in the area around the initial whole-cell breakthrough does not account for the observed changes in action potential dynamics. *Figure Contributions*: Emily Hill performed the experiments and analyzed the data.

Given a previous report that some populations of monomeric tau can readily seed aggregation to form toxic oligomers (Mirbaha et al., 2018), we decided to investigate whether tau protein monomers can induce changes in the action potential wave form or whether only oTau is responsible. Tau monomers (mTau; 444 nM, see Materials and Methods; Fig. 5*A*) were injected into hippocampal pyramidal cells. There was no significant change in the current−voltage relationship over time for neurons injected with mTau (Fig. 5*B*) and no change in action potential amplitude (at 40 min, the mean amplitude was 98.6% of the amplitude at time 0; Fig. 5*C*). It is therefore only the oligomeric form of tau that induces the changes in neuronal electrophysiological properties over a time-scale of ∼40 min. However, it is possible that at longer time scales the monomeric tau may oligomerize and then start to have effects on neuronal properties.

### The changes in action potential wave form cannot be accounted for by the aggregation of oTau increasing series resistance

The reduction in action potential amplitude and the slowing of rise and decay kinetics could be a result of electrotonic filtering due to an accumulation of oTau aggregates in the tip of the patch pipette increasing series resistance. This seems unlikely as there was no significant difference in series resistance between recordings made with vehicle in the patch pipette and with oTau (Fig. 5*D*). There was also no marked change in series resistance over the length of recordings when either oTau or vehicle were introduced into cells (Fig. 5*D*). To provide further confirmation, we conducted re-patching experiments (as in Fang et al., 2014; Fig 5*E–J*). Following whole-cell recording from a pyramidal cell for 20 min with either oTau (444 nm) or vehicle (control), the electrode was carefully removed from the cell surface and the cell was then re-recorded with an electrode which contained intracellular solution with vehicle (Fig. 5*E*,*J*). The changes in action potential kinetics induced by oTau were still present when cells were re-patched with pipettes containing intracellular with vehicle (Fig. 5*I*,*J*). The re-patching of cells which had originally been recorded with vehicle containing intracellular solution had no effect on action potential kinetics or amplitude (Fig. 5*F*,*G*). Therefore, the changes in action potential dynamics were not the result of oTau accumulating in the patch pipette or at the site of whole-cell breakthrough.

### Introduction of oTau into presynaptic cells markedly impairs basal synaptic transmission

**Figure 6.**
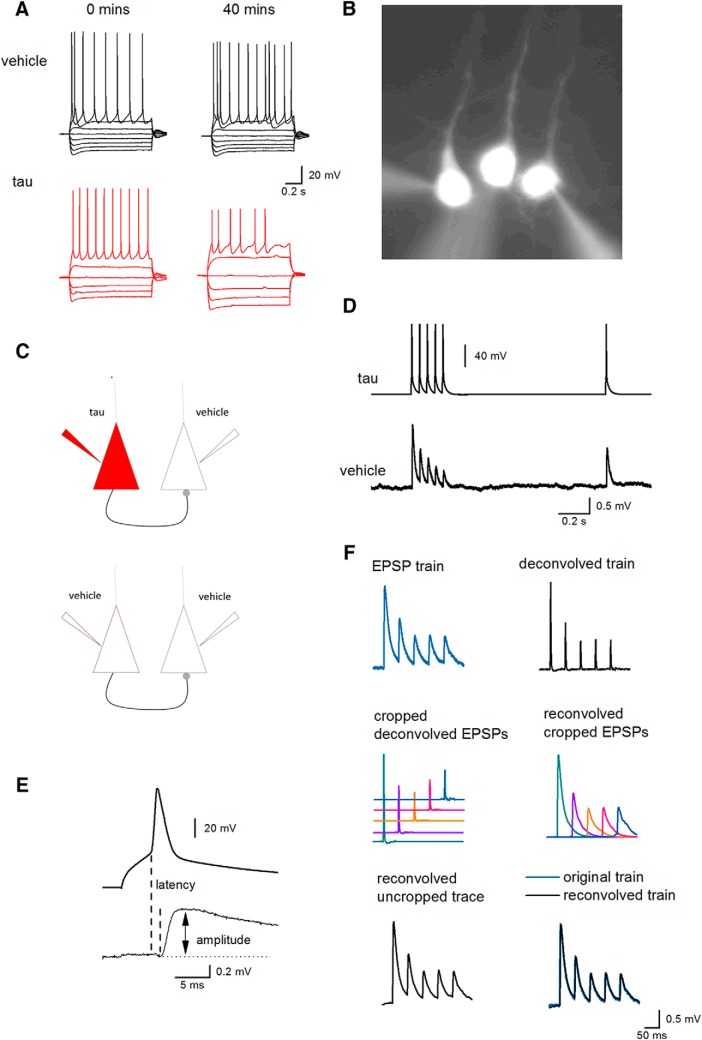
Protocols for testing the effect of oTau on synaptic transmission between thick-tufted Layer V somatosensory neocortical neurons. ***A***, Standard I-V relationships recorded from neocortical Layer V PCs using pipettes containing either vehicle (top panels) or oTau (666 nM, bottom panels). There was no significant change in the I-V relationship over 40 min in cells injected with vehicle, whereas oTau reduced action potential amplitude and increased cell input resistance. ***B***, Micrograph of three Layer V PCs labeled with Alexa Fluor 488. Recordings were routinely made from three neighboring pyramidal cells to increase the probability of finding synaptically connected cells. ***C***, Diagram of experimental protocol for synaptically connected pyramidal cells. Top panel, oTau was introduced into the presynaptic cell and vehicle was introduced into the postsynaptic cell. Bottom panel, For control recordings, vehicle was introduced into both presynaptic and postsynaptic neurons. ***D***, A train of five action potentials (20 Hz) and one recovery action potential were evoked in the presynaptic cell with short depolarizing current steps (top) and the resultant EPSPs were recorded in the postsynaptic cell (bottom). ***E***, Expanded traces of the first action potential (in the train) and the resultant EPSP illustrating the measurement of 1st EPSP amplitude and latency. ***F***, Method of accurately measuring the amplitude of the 2nd to 5th EPSPs in the train. EPSPs were averaged (after removing any baseline drift) and then deconvolved. The deconvolved EPSPs were cropped out and then reconvolved so that individual average EPSPs were not superimposed on the decay of the previous EPSP average. The accuracy of the deconvolution method was confirmed by reconvolving the EPSPs and comparing the resultant waveforms to the original untransformed EPSPs. *Figure Contributions*: Emily Hill performed the experiments and analyzed the data.

Given the marked of effects of oTau on action potential kinetics, the next step was to investigate if oTau introduction affected synaptic transmission. Due to the low connectivity between hippocampal pyramidal cells, between 1% and 5% (Debanne et al., 2008), the synapses between pairs of thick-tufted Layer V neocortical PCs were examined instead. Initially we confirmed that the introduction of oTau (444 nM) into Layer V PCs had similar effects on action potential properties to that observed in hippocampal CA1 pyramidal cells. Action potential amplitude was significantly reduced from 74.45 ± 2.13 mV at time 0, to 60.135 ± 6.38 mV after 40 min (*p* = 0.0137) in cells where oTau was injected (*n* = 8; Fig. 6*A*). There was no significant change in the amplitude of action potentials in neurons injected with vehicle (mean of 80.85 ± 1.82 mV at 0 min vs 77.87 ± 2.08 mV at 40 min, *p* = 0.1337, *n* = 10). Injection of oTau also significantly increased the input resistance (from 92.5 ± 7.58 MΩ at time 0 to 108.19 ± 8.107 MΩ at 40 min, *p* = 0.0446; Fig. 6*A*) with no difference following the introduction of vehicle (90.1 ± 11.95 vs 92.15 ± 12.99 MΩ, *p* = 0.672). Because the effects of oTau were slower in Layer V thick-tufted PCs, presumably due to their larger size, we used a larger concentration of oTau (666 nM) so that any changes in the properties of synaptic transmission occurred within a manageable time frame. To test whether oTau affected synaptic transmission, oligomer preparations were introduced into the presynaptic cell and vehicle was introduced into the postsynaptic cell (Fig. 6*C*). A train of five action potentials followed by a recovery action potential (1 s interval; Kerr et al., 2013; Fig. 5*D*) was used to test whether Layer V PCs were synaptically connected. Once the connectivity was verified, this stimulus was repeated (at an interval of 10 s) throughout the duration of recordings (up to ∼50–60 min). For the 1st EPSP, the amplitude and latency were measured from average EPSPs (Fig. 6*E*). Because of the small amplitude of unitary EPSPs (average amplitude ∼0.6 mV), average EPSPs (between 30 and 50 sweeps) were evaluated over the following time periods: 0–10, 10–20, 20–30, and 30–40 min. Deconvolution and then reconvolution enabled the accurate measurement of the amplitude of individual average EPSPs in the train in most recordings (Fig. 6*F*). However, in a small number of recordings the amplitude of the EPSPs in the train were so small that they could not be accurately resolved and thus these connections were only used for analysis of the 1st EPSP.

**Figure 7.**
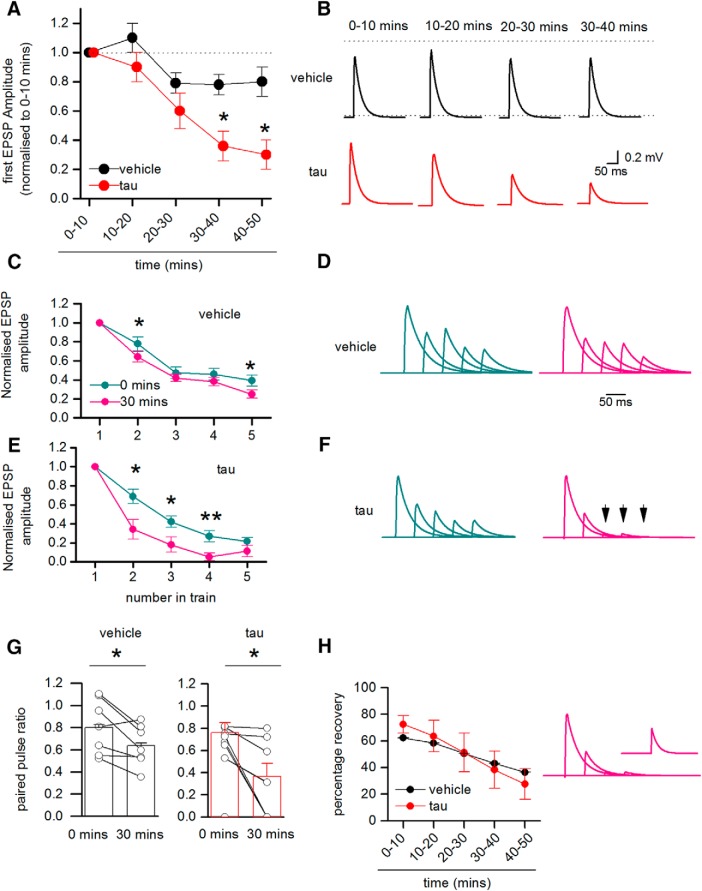
Presynaptic oTau impairs synaptic transmission between Layer V PCs. ***A***, The amplitude of the 1st EPSP in the train (relative to the amplitude of averaged EPSPs over the first 10 min of the recording) plotted against time for synaptically connected pairs with either vehicle or oTau (666 nM) introduced into the presynaptic cell. There was little change in the amplitude of EPSPs for connections where vehicle was introduced into presynaptic and postsynaptic cells (over 50 min), but there was a significant decline in the amplitude of EPSPs when oTau was introduced into the presynaptic cell (control vs tau 30–40 min *p* = 0.0293, 40–50 min *p* = 0.0344). ***B***, Example of averaged 1st EPSPs at different time points when either vehicle or oTau was introduced into the presynaptic cell. The EPSPs are averages of 30–50 sweeps and have been deconvolved, cropped and then reconvolved (as outlined in Fig. 6). ***C***, Graph plotting the amplitude of average EPSPs (relative to the amplitude of the 1st EPSP) against number in the train for 0–10 min of recording and for 30–40 min of recording, where vehicle was introduced into both presynaptic and postsynaptic cells. There was a small but significant increase in depression between time 0 and 30–40 min for the 2nd EPSP (*p* = 0.0316) and the 5th EPSP (*p* = 0.0409) in the train. ***D***, Example EPSP waveforms from a single recording when vehicle was introduced into both the presynaptic and postsynaptic cells. Averages were constructed at 0–10 min (left panel) and at 30–40 min (right panel) and are normalized so the amplitude of the 1st EPSP remains the same. There is little change in the degree of depression over time. ***E***, Graph plotting the average relative amplitude of EPSPs (relative to the 1st EPSP) against number in the train at 0–10 min and at 30–40 min with oTau present in the presynaptic cell. There was a significant increase in depression between 0 and 30–40 min for the 2nd EPSP 2 (*p* = 0.0347), 3rd EPSP (*p* = 0.0365), and 4th EPSP (*p* = 0.00941) in the train. ***F***, Example EPSP waveforms from a single recording when oTau was introduced into the presynaptic cell. Averages were constructed at 0–10 min (left panel) and at 30–40 min (right panel) and are normalized so the amplitude of the 1st EPSP remains the same. The 3rd to 5th EPSPs (arrows) are absent in the 30- to 40-min average although the action potentials did not fail in the presynaptic cell. ***G***, Graphs plotting paired pulse ratio (amplitude of 2nd EPSP/amplitude of 1st EPSP) for cells with vehicle present and for recordings when oTau was introduced into the presynaptic cell. The points are means from single experiments and the bars show the mean and SEMs for all recordings. In both treatments there was a significant fall in the paired-pulse ratio over the duration of the recording (vehicle *p* = 0.0316 and oTau *p* = 0.0347). ***H***, Graph plotting the percentage recovery in EPSP amplitude measured by evoking an EPSP 1 s after the end of the train. If there was 100% recovery, then the recovery EPSP has the same amplitude as the 1st EPSP. The amount of recovery decreases over time and is similar for cells with either vehicle or oTau introduction. Right panel, Waveforms after 30–40 min (same recording as in ***F***) showing the absence of the 3rd to 5th EPSPs, but there is some recovery in transmission after a 1-s interval (inset shows mean recovery EPSP). *P < 0.05, **P < 0.01.*Figure Contributions*: Emily Hill performed the experiments and analyzed the data.

Out of 230 paired recordings, 25 pairs of neurons were synaptically connected (one in 9.2 pairs). In 10 of the connected pairs, vehicle was introduced into both the presynaptic and postsynaptic cells (as a control). In another 10 connections, oTau was introduced into the presynaptic cell and vehicle was introduced into the postsynaptic cell, and in a further four recordings, oTau was introduced into the postsynaptic cell and vehicle in the presynaptic cell. At early time points (0–10 min), there was no significant difference in the amplitude of the 1st EPSP in the train (*p* = 0.51) or its latency (*p* = 0.581) when either vehicle or oTau were introduced into the presynaptic cell (mean unitary EPSP amplitude: vehicle 0.75 ± 0.13 mV; oTau 0.61 ± 0.13 mV; latency: vehicle 2.2 ± 0.0002 ms, oTau 2.1 ± 0.0001 ms). When vehicle was introduced into both the presynaptic and postsynaptic cells, the amplitude of the 1st EPSP in the train remained relatively stable for the duration of recordings (the amplitude of the 1st EPSP after 40–50 min of recording was 90.5 ± 14% of the EPSP amplitude at 0–10 min of recording). However, when oTau was introduced into the presynaptic cell, although the recordings remained stable (mean membrane potential at time 0, 67.5 ± 1.5 mV vs membrane potential 40 min, 62.5 ± 1.3 mV), the amplitude of the 1st EPSP in the train was markedly diminished in seven out of nine of the recordings. At 40–50 min, the amplitude of the 1st EPSP was significantly (*p* = 0.034) reduced to 30 ± 10% of the amplitude of EPSPs at 0–10 min (*n* = 9; Fig. 7*A*,*B*). This reduction in EPSP amplitude was not a result of failed action potential firing in the presynaptic cell, as the small number of sweeps in which any of the action potentials in the train failed (46 out of 3900 sweeps, ∼1.2%) were excluded from analysis. This indicates that the introduction of oTau into the presynaptic cell leads to a marked impairment in synaptic transmission between Layer V pyramidal neurons that occurs within a short time frame.

### The deficits in synaptic transmission are associated with increased short-term depression

We next examined whether short-term synaptic plasticity was altered by oTau by measuring the degree of depression induced by firing five action potentials at 20 Hz (50-ms interval). If the reduction in EPSP amplitude induced by oTau is a consequence of a fall in the probability of transmitter release, then short-term depression may be reduced (equivalent to the activation of presynaptic receptors such as adenosine A_1_; Kerr et al., 2013). For analysis, the amplitude of EPSPs in the train (2nd to 5th EPSP) were measured relative to the amplitude of the 1st EPSP. In 9 out of 10 recordings (in one recording the connection was too weak to accurately measure the amplitude of the 2nd to 5th EPSPs) with vehicle introduced into both the presynaptic and postsynaptic cells, there was little change in the degree of short-term depression over the duration of the recordings (Fig. 7*C*,*D*). There was a small (but significant) increase in depression as there was a decrease in the paired-pulse ratio (2nd EPSP/1st EPSP amplitude) over 40 min (Fig. 7*G*). In the recordings (eight out of nine, one connection was too weak to accurately measure) where oTau was introduced into the presynaptic cell, there was an increase in the degree of short-term depression (Fig. 7*E*,*F*). In four out of the eight connections, there was a failure of transmission: the 2nd to 5th EPSPs were absent (Fig. 7*E*). This was not a result of failed action potential firing, as any failures of presynaptic firing were excluded from analysis (as outlined above). There was also a significant decrease in the paired-pulse ratio (1st EPSP/second EPSP), but this was comparable to the connections with vehicle in presynaptic and postsynaptic cells. To examine the duration of the depression, we measured the relative amplitude of the recovery EPSP, which was evoked 1 s after the train of action potentials (Fig. 7*H*). Although there was greater depression in connections with oTau present in the presynaptic cell, the recovery (amplitude of recovery EPSP/1st EPSP amplitude) was not different to connections with vehicle introduced into presynaptic and postsynaptic cells. This indicates that the additional short-term depression induced by oTau recovers over the same period as the depression in control conditions. Therefore, the impairment of synaptic transmission is associated with an increase in depression and thus differs from the activation of presynaptic receptors.

### Introducing oTau into the postsynaptic cell does not impair basal synaptic transmission

**Figure 8.**
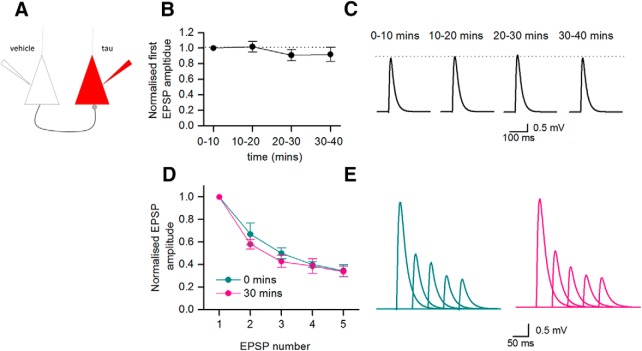
Introduction of oTau into postsynaptic neurons has no significant effect on basal synaptic transmission. ***A***, Diagram of experimental protocol: vehicle was introduced into the presynaptic cell and oTau (666 nM) was introduced into the postsynaptic cell. ***B***, The amplitude of the 1st EPSP in the train (relative to the amplitude of averaged EPSPs over the first 10 min of the recording) plotted against time with oTau present in the postsynaptic cell. There was no significant change in the amplitude of the 1st EPSP amplitude in the train for the duration of recordings. ***C***, Examples of averaged EPSPs (first in train) at different time points throughout the recording (oTau present in the postsynaptic cell). The EPSP averages have been deconvolved, cropped, and then reconvolved (as in Fig. 6). ***D***, The mean amplitude of the 2nd to 5th EPSPs in the train normalized to the amplitude of the 1st EPSP in the train, averaged at 0–10 min and at 30–40 min. There was no significant change in the degree of short-term depression over that time period. ***E***, Average EPSP waveforms from a single recording where oTau was introduced into the postsynaptic cell. Averages were constructed at 0–10 min (left panel) and at 30–40 min (right panel) and are not normalized. *Figure Contributions*: Emily Hill performed the experiments and analyzed the data.

In four connected pairs of Layer V PCs, oTau was introduced into the postsynaptic cell with vehicle introduced into the presynaptic cell (Fig. 8*A*). In these connections, there was no significant reduction in the amplitude of the 1st EPSP in the train over time (average EPSP amplitude after 40 min was 91.8% of the EPSP recorded at 0–10 min; Fig. 8*B*,*C*), and there was little change in short-term depression over time (Fig. 8*D*,*E*). Unlike the marked effects observed when oTau was introduced into the presynaptic cell, introduction of oTau into the postsynaptic neuron did not impair basal synaptic transmission over the duration of the recordings.

### Postsynaptic oTau injection impairs LTP

**Figure 9.**
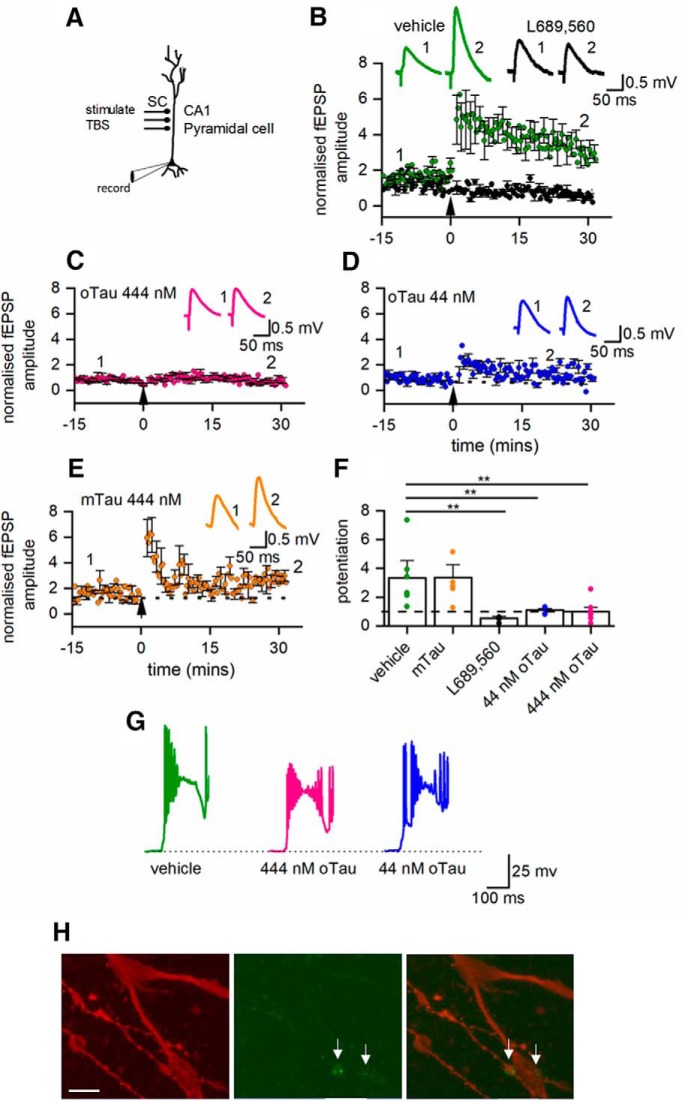
Introduction of oTau into the postsynaptic neuron blocks the induction of LTP. ***A***, Diagram of experimental methods to measure LTP. EPSPs were recorded in CA1 hippocampal pyramidal cells in response to Schaffer collateral stimulation. LTP was induced by TBS. ***B***, Mean EPSP amplitude plotted against time for seven slices in control (vehicle introduced into pyramidal cells) and in three slices where the NMDA receptor antagonist L689,560 (5 μM) was present. TBS evoked robust potentiation in control conditions which was abolished by L689,560. Inset, Average waveforms before (1) and 30 min after (2) TBS in control conditions and in the presence of L689,560 (5 μM). ***C***, Mean EPSP amplitude plotted against time for seven slices with oTau (444 nM) present in the intracellular recording solution. LTP was abolished. Inset, Average waveforms before (1) and 30 min after (2) TBS. ***D***, Mean EPSP amplitude against time for five slices with oTau (44 nM) present in the intracellular recording solution. LTP was abolished but there is some short-term potentiation. Inset, average waveforms before (1) and 30 min after (2) TBS. ***E***, Graph plotting mean EPSP amplitude against time for five slices with monomeric Tau (444 nM) present in the intracellular recording solution. LTP was induced in the presence of monomeric Tau. Inset, average waveforms before (1) and 30 min after (2) TBS. ***F***, Summary. In control conditions (vehicle in the intracellular solution) after 30 min following TBS, EPSP amplitude was potentiated to 3.31 ± 1.32× baseline amplitude (*n* = 7). This potentiation was lost in the presence of L689,560 (0.53 ± 0.14, *n* = 3), with oTau 444 nM in the intracellular solution (0.989 ± 0.31, *p* = 0.042, *n* = 7, control vs 444 nM) and with 44 nM oTau in the intracellular solution (1.11 ± 0.1, *n* = 5). In contrast potentiation persisted with mTau (444 nM) in the intracellular solution (3.35 ± 0.90, *n* = 5). One-way ANOVA gives a significant effect of treatment (*F*_(4,22)_ = 5.645, *p* = 0.0028) and Fisher *post hoc* comparisons shows significant differences between the potentiation of cells injected with vehicle and L689,560, 44, or 444 nM oTau (*p* = 0.0055, *p* = 0.0077, and *p* = 0.0018, respectively). ***G***, Example voltage responses to the first burst in the TBS for a neuron where vehicle was introduced, where 444 nM oTau was introduced and where 44 nM oTau was introduced. ***H***, Photomicrographs of the dendrite bifurcation from a labeled hippocampal neuron where 444 nM oTau was introduced at the soma. Left panel, Dendrites labeled with Alexa Fluor 594 introduced from patch pipette. Middle panel, Fluorescent puncta of oTau. Right panel, Merged image showing dendrites (red) and oTau puncta (green). Scale bar = 12 μM. *P < 0.01.*Figure Contributions*: Emily Hill performed the experiments and analyzed the data.

Although introduction of oTau into the postsynaptic neuron had no effect on basal synaptic transmission it may modulate synaptic plasticity, as it has been suggested that the inhibitory effects of extracellular oTau on LTP are postsynaptic (Ondrejcak et al., 2018). To investigate this possibility, we made whole-cell current-clamp recordings from CA1 hippocampal neurons and stimulated Schaffer collaterals (Fig. 9*A*). We took this approach as access to presynaptic neurons was not required and it is much more straightforward to carry out the experiment in hippocampal slices. oTau, vehicle, or mTau was introduced into the postsynaptic neuron during current-clamp recording and the effects on LTP measured. There was no significant difference in the amplitude of EPSPs across all of the conditions at time 0 (one-way ANOVA, *p* = 0.14783). When vehicle was introduced into the postsynaptic cell, TBS induced robust potentiation (30 min after the stimulation, EPSP amplitude was 3.31 ± 1.32× the baseline amplitude, *n* = 7; Fig. 9*B*,*F*). This potentiation was NMDA receptor-dependent as it was abolished by the NMDA receptor antagonist L689,560 (5 μM, 0.53 ± 0.14× the baseline EPSP amplitude after 30 min, *n* = 3; Fig. 9*B*,*F*). In initial experiments, we found that oTau (444 nM) abolished both short term potentiation (STP) and LTP (*n* = 7 slices; Fig. 9*C*,*F*). The experiments were repeated with a 10-fold reduction in the concentration of oTau (44 nM), which also abolished LTP but did not prevent STP (*n* = 5; Fig. 9*D*,*F*). Monomeric tau (444 nM) had little effect on the degree of potentiation (LTP or STP, *n* = 5; Fig. 9*E*,*F*). This indicates that the presence of oTau in postsynaptic cells will interfere with synaptic plasticity which could relate to early changes in learning behavior. One possible mechanism for this interference with plasticity is a change in the voltage response to the TBS leading to a subthreshold depolarization. To investigate this possibility, we examined the TBS voltage responses and found that there was large variability in response to TBS within control recordings (vehicle in the postsynaptic cell). There were some responses where the postsynaptic cell did not fire, where the cell fired repeatedly across all the bursts and where the cell only fired in response to the first burst. However, robust LTP was produced in all cases. We observed a similar range of responses across all of the conditions, with no correlation between LTP size and firing pattern (Fig. 9*G*). Therefore, it appears unlikely that a reduction in stimulus-response can account for the loss of LTP and also because the oTau is only in the postsynaptic cell it can be accounted for by impairment of basal synaptic transmission.

The effects of oTau on synaptic transmission and LTP strongly suggest that oTau must reach synaptic locations. To investigate this, we fully reconstructed a subset of four hippocampal neurons in which 444 nM oTau was introduced into the soma via a patch pipette and recordings were made for 20 min. In these neurons, puncta of oTau could clearly be observed in the distal dendrites (up to the bifurcation) and could also be observed in the axon (Fig. 9*H*). This indicates that the introduced oTau can spread from the soma into dendritic and axonal compartments in ∼20 min and is consistent with the effects on neurophysiology.

## Discussion

This is the first study to investigate the effects of introducing oligomeric tau protein (oTau) directly into the soma of mammalian neurons in acute brain slices and then using whole-cell patch clamp recording to investigate the resultant changes in electrophysiological properties and synaptic transmission.

In several previous studies, tau oligomers have been applied to brain slices and neuronal cultures via the extracellular media (Lasagna-Reeves et al., 2012; Booth et al., 2016; Fá et al., 2016; Puzzo et al., 2017). Although tau has clear effects in these studies, it is difficult to determine whether tau is acting on the outside of neurons, to quantify how much tau is taken up into neurons and whether the observed effects on synaptic transmission are pre or postsynaptic. Transgenic models of tauopathies have also been used to investigate changes in neuronal properties produced by tau (Rudinskiy et al., 2014; Zhou et al., 2017; McInnes et al., 2018). In these studies, it is difficult to measure the concentration of oTau present and the specific oTau conformers that mediate the toxicity. The successful use of patch clamp electrodes to introduce characterized α-synuclein oligomers into mammalian neurons has been published previously (Kaufmann et al., 2016). Here, using the same strategy, we have measured the time-dependent and concentration-dependent effects of oTau. A major difference between our study and previous studies (apart from the method of introducing tau oligomers) is that only one neuron in a network will be affected by the introduced tau. This means that any changes in electrophysiological properties that are measured are direct and do not arise from compensatory changes in networks that may occur if the properties of many neurons are affected. Other major advantages of our approach include using the neuron (at whole-cell breakthrough) as an internal control for each recording and the removal of any slow cellular uptake steps which maybe present when tau is applied extracellularly. Furthermore, this method only requires a small amount of oTau and allows the targeting of oTau into either pre or postsynaptic neurons.

We used a method to prepare oTau which did not involve any inducers (such as heparin, RNA, etc.), as they may potentially produce cellular effects themselves. We have used Alexa Fluor 488 to label and also to limit the aggregation to oTau into fibrils. While an obvious caveat to this method is that we have mutationally altered tau-441, CD spectral analysis shows the mutations and labeling have only minor effects on tau structure (Fig. 1*E*). More importantly, we have now established a proof of principle in the successful performance of these studies that in the future can be extended for in-depth analysis of different oTau preparations including tau with clinically relevant mutations. The oTau we prepared had a granular/spherical structure and a predominant β-sheet conformation (Fig. 1). The oTau reacted strongly to the T22 oligomeric tau antibody, the HT7 mid-region tau antibody and the K9JA tau repeat domain-binding antibody. We have confirmed that the tau preparations used for electrophysiological analysis were oligomeric, bearing the aggregation-defining repeat domain and a clinically relevant mid-region epitope used in biomarker tests for tauopathies (Chen et al., 2019). The tau oligomers were diluted into the intracellular solution after filtering to prevent binding to the filter and loss of protein (Fig. 1). Clearly the tau could oligomerize *in vitro*, although it was not in a hyperphosphorylated state. In contrast hyperphosphorylation has been implicated in the oligomerization process that occurs *in vivo* (Avila et al., 2006; Qiang et al., 2018). Taken together this suggests that the hyperphosphorylation may be required for the dissociation of tau from the microtubules *in vivo* but not for the oligomerization step.

One interesting question is what is the phosphorylation state of the tau oligomers once they have been introduced into the neurons? Since the oligomers were un-phosphorylated before their introduction into neurons, any changes in the phosphorylation status post introduction (whether to the introduced oligomers or endogenous tau) might play a role in the mechanism of the observed effects. Experiments on phospho-tau status are often reported using Western blottings and densitometry. However, this is not a feasible way to determine tau phosphor status, using our single cell introduction method. More recently new mass spectrometric assays have been developed (Barthélemy et al., 2019) but, again, require substantial material to determine changes in phosphor status. Furthermore, the full-length oligomers used in this study contain around 80+ epitopes that can be phosphorylated. While specific phospho-Ser/Thr/Tyr antibodies are available for several of these, it is unlikely that a single cell immunofluorescence analysis will yield interpretable results.

### The effects of tau oligomers are not due to accumulation of aggregates in pipette or in area of whole-cell breakthrough

A number of control experiments were used to validate that the effects of oTau introduction were specific and not an artifact of the recording and introduction protocol. There was no significant difference in the electrophysiological parameters following the introduction of vehicle (buffer without tau) or the addition of a similar size aggregation-competent molecule, BSA (20 μM). It is conceivable that either the narrow tip of the patch-pipette becomes clogged with aggregated tau or tau accumulates in the soma around the breakthrough site leading to filtering of the signals (particularly the action potential). Three pieces of experimental evidence makes this seem unlikely. Firstly, there was no difference in either the initial series resistance across the conditions and no significant increase in series resistance for the duration of the recordings (Fig. 5*D*). Secondly, in re-patching experiments, the changes in action potential amplitude and kinetics persisted when the cells were re-patched with intracellular solution containing vehicle. Finally, when oTau was introduced into the postsynaptic cell, it had no effect on the amplitude or kinetics of the recorded EPSPs. If the effects of oTau were simply a filtering effect, then the EPSP amplitude would be expected to decrease and EPSPs would also have slower rise and decay kinetics. It has been shown before that α-synuclein oligomers (at a similar concentration, 500 nM), which were injected into cortical neurons, had no effect on action potential amplitude and the action potential rise and decay kinetics were not slowed (Kaufmann et al., 2016). This suggests that the introduction of aggregating oligomeric species does not produce generalized filtering effects per se.

### Tau oligomers modify action potential amplitude and kinetics but have little effect on subthreshold properties

Using standard step and dynamic current−voltage responses, we found that oTau had little effect on the majority of the subthreshold parameters that were measured. It was only at late time points (after 40 min) and with the larger concentrations of oTau (444–666 nM) that significant changes in input resistance (increased) and membrane potential (depolarized) occurred leading to an increased action potential firing rate. It may be that lower concentrations of oTau (44–133 nM) would eventually have effects on input resistance and membrane potential if the recordings lasted long enough (a small increase in firing rate was observed at late time points for six out of seven cells with 133 nM oTau). An increase in input resistance could result from the block of a standing (or leakage) conductance or possibly from the electronic conversion of the cell from multi compartments to a single compartment by reducing current flow. The oTau-induced depolarization is consistent with previously reported data from the rTg4510 mouse model, which expresses human tau variant P301L, where the pyramidal cells are depolarized by ∼8 mV compared to WT littermates (Rocher et al., 2010).oTau markedly altered the action potential wave form causing a slowing of rise and decay kinetics and a reduction in peak amplitude. These effects were concentration dependent, occurring more rapidly with larger concentrations of oTau and did not occur with tau monomers. The mechanism underlying these changes in action potential wave form are currently not clear. It is possible that the aggregation of oTau in the soma act as a barrier to current flow from the initial segment in the axon (where Na^+^ channels are concentrated) to where the action potential is recorded in the soma or it could be due to changes in voltage gated Na^+^ or K^+^ channels.

### Presynaptic tau oligomer injection impairs synaptic transmission

The introduction of oTau into the presynaptic neuron led to a large reduction in synaptic strength, an effect which was not observed when oTau was introduced into the postsynaptic neuron or if vehicle was introduced into both presynaptic and postsynaptic neurons. An inhibition of synaptic transmission has been previously reported in transgenic tau models (Zhou et al., 2017; McInnes et al., 2018). The inhibition of transmitter release by the activation of presynaptic receptors (such as adenosine A_1_ or GABA_B_) is associated with an increase in paired-pulse ratio and a reduction in depression across a train of EPSPs (Kerr et al., 2013). This contrasts with the inhibitory effects of oTau which were not associated with a decrease in depression across a train of stimuli. In many of the recordings, the later EPSPs in the train failed, although there was some recovery of transmission following a 1-s interval. It has been reported in synapses between cultured hippocampal neurons from transgenic mice expressing tau that synaptic depression is enhanced and that this results from the crosslinking of vesicles with actin to slow vesicle movement (Zhou et al., 2017). Our data are consistent with a reduction in the rate of vesicle restock although this does not account for the change in the first EPSP.

Does the change in action potential wave form contribute to the inhibition of synaptic transmission? Injection of human tau into the squid axon has been shown to result in inhibition of synaptic transmission without changing the action potential wave form Moreno et al. (2016), suggesting that tau can directly affect the release machinery in the synaptic terminal. It is possible that the changed dynamics of the action potential wave form interferes with propagation down the axon to the release sites, leading, for example, to branch-point failure. This, however, seems unlikely, as it has been previously shown that complex spikes consisting of two to three full sized action potentials followed by several highly attenuated “spikelets” are all fully propagated down the axon of CA1 pyramidal cells (Apostolides et al., 2016).

### Postsynaptic oTau disrupts LTP

Introduction of either 44 or 444 nM oTau into postsynaptic hippocampal neurons prevented the induction of LTP, with 444 nM also abolishing short-term potentiation. These effects were not observed when either vehicle or monomer (444 nM) was introduced into postsynaptic neurons. Previous studies have shown that extracellular oTau can impair LTP (Lasagna-Reeves et al., 2012; Fá et al., 2016) with monomeric tau inactive. In these experiments, oTau was introduced via the extracellular medium and thus it is unclear whether the site of action was either presynaptic or postsynaptic or there was activity at both sites. Ondrejcak et al. (2018) demonstrated that for a subset of AD brain extracts, that produced amyloid β-independent reductions in LTP, immunodepletion of the extract with the Tau5 monoclonal antibody prevented the impairment of LTP. These studies provide a strong argument for testing the effect of direct neuronal oTau introduction on LTP. Our study validates the results of previous studies and also provides direct evidence that the postsynaptic effects of oTau are sufficient to abolish LTP. The observed defects in LTP could result from oTau increasing the threshold for LTP induction or by blocking LTP induction mechanisms. The former could be the result of oTau changing the voltage response to the TBS so that insufficient depolarization reaches the dendritic spines to remove the NMDA receptor Mg^2+^ block. Although this possibility cannot be completely ruled out, it appears unlikely as there were no consistent changes in TBS voltage responses and the responses were highly variable under control conditions. Recent experiments have highlighted the importance of tau in plasticity (Regan et al., 2015) with its correct phosphorylation required for long-term depression. It is possible that the introduced oTau could bind or sequester the tau present in the dendrites leading to inhibition of plasticity.

Following whole-cell recordings (up to 40- to 50-min duration), fluorescent oTau could be clearly observed in both the cell body of CA1 hippocampal pyramidal cells and also in the distal dendrites and axons. This is consistent with the observed effects of oTau on synaptic transmission and plasticity.

### Concluding statement

Using a targeted approach, we have introduced oTau into cortical neurons and have shown rapid changes in electrophysiological properties, synaptic transmission and synaptic plasticity.

## Synthesis

Reviewing Editor: Jun Chen, University of Pittsburgh Decisions are customarily a result of the Reviewing Editor and the peer reviewers coming together and discussing their recommendations until a consensus is reached. When revisions are invited, a fact-based synthesis statement explaining their decision and outlining what is needed to prepare a revision will be listed below. The following reviewer(s) agreed to reveal their identity: Efthimios Skoulakis, Karunya Kandimalla.

Both reviewers acknowledged that this is a well-performed study and the results are potentially important, especially for the tauopathy research field. Both reviewers have some specific suggestions on improving the manuscript.

Reviewer #1

This is a strong manuscript detailing a novel approach that has yielded significant and informative results.

The main concern this reviewer has is that the protein used to produce the reported results contains multiple mutations, atypical for most Tauopathies (except perhaps FTDP-17?). This reviewer does understand the significance of ascertaining oligomerization, but these the mutated cysteines may not simply block aggregation. There are methods to enrich a Tau preparation in oligomers. Would such a preparation at a high concentration recapitulate one or more of the main findings (ie the decline in amplitude?)

Another issue is the phosphorylation state of Tau. Hyperphosphorylated Tau is typically associated with Tauopathies and the pathologies have come to be closely linked to pathologies. In addition, a typical phosphorylation pattern would argue against unpredictable effects of the cysteine mutations.

Finally, this reviewer recommends a thorough language editing of the manuscript to smoothen rough spots such as that at the end of the first paragraph on page 18 with so many “thus” in two sentences

“Thus, the additional short-term depression induced by oTau recovers over the same period as the depression in control conditions. Thus the impairment of synaptic transmission is associated with an increase in depression and thus differs from the activation of presynaptic receptors”

Reviewer #2

The authors should report standard deviation instead of standard error of the mean, which does not capture the biological variability among the samples.

## Author Response

We thank the reviewers for their insightful comments

We have added one new piece of data to the manuscript which we feel strengthens the observed electrophysiological changes produced by oTau. Using the Zeiss Airyscan module we have fully reconstructed a small number of neurons and are able to resolve oTau puncta within the dendrites (upto the bifurcation) and in the axon. As these are the locations of synapses this fits with the observed changes in synaptic transmission and plasticity. We have included some micrographs in figure 9.

Referee 1

The main concern this reviewer has is that the protein used to produce the reported results contains multiple mutations, atypical for most Tauopathies (except perhaps FTDP-17?). This reviewer does understand the significance of ascertaining oligomerization, but these the mutated cysteines may not simply block aggregation. There are methods to enrich a Tau preparation in oligomers. Would such a preparation at a high concentration recapitulate one or more of the main findings (ie the decline in amplitude?)

We have used full-length human Tau-441 which contains no mutations responsible for early onset tauopathies. We have deliberately taken the approach of not using any methods to enrich oligomerisation (ie Heparin etc) as they may have effects when introduced into cells. The mutation of cysteines had 2 purposes 1) is to allow labelling and 2) to actually reduce the extent of oligmerisation/fibrilisation. We have added additional data (figure 1) to show that the secondary structure is very similar for oTau without changed cysteines vs oTau with changed cysteines, suggesting that both had similar beta sheet conformations which drive aggregation. In addition, several groups (see below) have used oTau containing the same cysteine modifications to study multiple aspects of tau aggregation, inter-cellular propagation and cytotoxicity.

1. Kumar, S., Tepper, K., Kaniyappan, S., Biernat, J., Wegmann, S., Mandelkow, E.-M., Müller, D. J., and Mandelkow, E. (2014) Stages and Conformations of the Tau Repeat Domain during Aggregation and Its Effect on Neuronal Toxicity. J. Biol. Chem. 289, 20318-20332 2. Manger, L. H., Foote, A. K., Wood, S. L., Holden, M. R., Heylman, K. D., Margittai, M., and Goldsmith, R. H. (2017) Revealing Conformational Variants of Solution-Phase Intrinsically Disordered Tau Protein at the Single-Molecule Level. Angew. Chem. Int. Ed. 56, 15584-15588 3. Margittai, M., and Langen, R. (2004) Template-assisted filament growth by parallel stacking of tau. Proc. Natl. Acad. Sci. U. S. A. 101, 10278-10283 4. Michel, C. H., Kumar, S., Pinotsi, D., Tunnacliffe, A., George-Hyslop, P. S., Mandelkow, E., Mandelkow, E.-M., Kaminski, C. F., and Schierle, G. S. K. (2014) Extracellular Monomeric Tau Protein Is Sufficient to Initiate the Spread of Tau Protein Pathology. J. Biol. Chem. 289, 956-967 5. Shammas, S. L., Garcia, G. A., Kumar, S., Kjaergaard, M., Horrocks, M. H., Shivji, N., Mandelkow, E., Knowles, T. P. J., Mandelkow, E., and Klenerman, D. (2015) A mechanistic model of tau amyloid aggregation based on direct observation of oligomers. Nat. Commun. 10.1038/ncomms8025

We thank reviewer 1 for their thoughts on the phosphorylation pattern of our injected oligomers and agree with the hypothesis that a normal phosphorylation pattern would provide

good supporting evidence. We believe however that the rapid nature of the electrophysiological effects we see, the lack of data in the literature on the rapidity of phosphorylation and the extent of potential phosphorylation targets, make such an analysis beyond the scope of our current study.

To detail:

1. The full length tau oligomers were prepared in vitro and are un-phosphorylated before injection 2. We don't know the phosphorylation status of the oligomers once they are introduced into neurons, and how they might change, within the short time frame of these experiments.

Indeed, we are unaware of any published details of such very rapid, single cell, phospho-tau assays. Suo et al (2003) published that within 24 hours both PAR4 and MAPK can induce tau phosphorylation in hippocampal cell lines, when stressed by thrombin. Experiments on phospho-tau status are often reported using Western blots and densitometry. Using our single cell injection methods this is not a feasible way to determine tau phospho-status here. More recently new mass spectrometric assays have been developed, Barthélemy et al (2019), but again require substantial material to determine changes in status. The full length oligomers used in our study contain around 80+ epitopes that can be phosphorylated . While specific phospho-Ser/Thr/Tyr antibodies are available for several of these, it is unlikely that a single cell immunofluorescence analysis will yield interpretable results. For example, Whiteman et al (2011) have demonstrated some rapid changes (60 minutes) by western analysis of several phospho-epitopes including the microtubule binding domain epitope identified by 12E8, as being dependent on the energy status in cell culture. While suggesting it can be rapid - this study does not address the effects on oligomers, but rather tau released from presumably destabilising microtubules. While the western analysis is convincing, the largely qualitative interpretation of the micrographs is difficult for such time course experiments and in their case 12E8 was differentially increased while all others assayed decreased. The complexities of the tau phospho-code would make such analyses an extensive separate study (Kimura et al, 2018).

The importance of phosphorylation in other aspects of tau biology has however recently been shown by Hu et al (2016). They demonstrated that stereotactic injection of phosphorylated soluble oligomers isolated from AD brains increased the movement (the seeding) of tau from cell to cell in a particular humanised transgenic mouse model. This was restricted if the tau preparations were dephosphorylated before injection. However, while interesting in terms of the prion-like spread mechanism, the early functional consequences are unknown.

We consider that the rapid electrophysiological changes reported here may well be dependent on a phosphorylation dependence of the oligomers, and we will be analysing such changes in future experiments. Since the oligomers were un-phosphorylated pre-injection, any changes in the phosphorylation status post-injection (whether to the introduced oligomers or endogenous tau) are likely to play a role in the mechanism of the observed effects. However, we did not explore the mechanism here, thus we feel this comment is out of scope of our current study. We thank the reviewer again for their insight.

We have added some of this material to the discussion.

Barthélemy NR et al (2019) Tau Phosphorylation Rates Measured by Mass Spectrometry Differ in the Intracellular Brain vs. Extracellular Cerebrospinal Fluid Compartments and Are Differentially Affected by Alzheimer's Disease. Front Aging Neurosci. 2019 May 21;11:121. doi: 10.3389/fnagi.2019.00121

Hu W, et al. Hyperphosphorylation determines both the spread and the morphology of tau pathology. Alzheimers Dement. 2016;12(10):1066-1077.

Kimura T et al. (2018) Phospho-Tau Bar Code: Analysis of Phosphoisotypes of Tau and Its Application to Tauopathy. Front Neurosci. 6;12:44. doi: 10.3389/fnins.2018.00044

Suo Z et al. (2003) Rapid tau aggregation and delayed hippocampal neuronal death induced by persistent thrombin signaling. J Biol Chem. 278(39):37681-9.

Whiteman et al . (2011) Rapid changes in phospho-MAP/tau epitopes during neuronal stress: cofilin-actin rods primarily recruit microtubule binding domain epitopes. PLoS One. 6(6):e20878. doi: 10.1371/journal.pone.0020878

We have modified the text to remove the overuse of the term thus.

Reviewer 2

We have added the standard deviations to Table 1 and have produced a table of all the mean and standard deviations present in the manuscript which we have added into the supplementary material.

## References

[B1] Andorfer C, Kress Y, Espinoza M, De Silva R, Tucker K, Barde Y, Duff K, Davies P (2003) Hyperphosphorylation and aggregation of tau in mice expressing normal human tau isoforms. J Neurochem 86:582–590. 10.1046/j.1471-4159.2003.01879.x 12859672

[B2] Apostolides P, Milstein A, Grienberger C, Bittner K, Magee J (2016) Axonal filtering allows reliable output during dendritic plateau-driven complex spiking in CA1 neurons. Neuron 89:770–783. 10.1016/j.neuron.2015.12.040 26833135

[B3] Avila J (2010) Intracellular and extracellular tau. Front Neurosci 4 10.3389/fnins.2010.00049 PMC2907258 20661459

[B4] Avila J, Santa-María I, Pérez M, Hernández F, Moreno F (2006) Tau phosphorylation, aggregation, and cell toxicity. J Biomed Biotechnol 2006:74539. 10.1155/JBB/2006/74539 17047313 PMC1479889

[B5] Badel L, Lefort S, Brette R, Petersen CCH, Gerstner W, Richardson MJE (2008) Dynamic I-V curves are reliable predictors of naturalistic pyramidal-neuron voltage traces. J Neurophysiol 99:656–666. 10.1152/jn.01107.2007 18057107

[B6] Barthélemy NR, Mallipeddi N, Moiseyev P, Sato C, Bateman RJ (2019) Tau phosphorylation rates measured by mass spectrometry differ in the intracellular brain vs. extracellular cerebrospinal fluid compartments and are differentially affected by Alzheimer's disease. Front Aging Neurosci 11:121. 31178717 10.3389/fnagi.2019.00121 PMC6537657

[B8] Bezanson J, Edelman A, Karpinski S, Shah V (2017) Julia: a fresh approach to numerical computing. SIAM Rev 59:65–98. 10.1137/141000671

[B9] Booth C, Witton J, Nowacki J, Tsaneva-Atanasova K, Jones M, Randall A, Brown J (2016) Altered intrinsic pyramidal neuron properties and pathway-specific synaptic dysfunction underlie aberrant hippocampal network function in a mouse model of tauopathy. J Neurosci 36:350–363. 10.1523/JNEUROSCI.2151-15.2016 26758828 PMC4710765

[B10] Castillo-Carranza D, Guerrero-Munoz M, Sengupta U, Hernandez C, Barrett A, Dineley K, Kayed R (2015) Tau immunotherapy modulates both pathological tau and upstream amyloid pathology in an Alzheimer's disease mouse model. J Neurosci 35:4857–4868. 10.1523/JNEUROSCI.4989-14.2015 25810517 PMC6705372

[B11] Chen Z, Mengel D, Keshavan A, Rissman R, Billinton A, Perkinton M, Percival-Alwyn J, Schultz A, Properzi M, Johnson K, Selkoe D, Sperling R, Patel P, Zetterberg H, Galasko D, Schott J, Walsh D (2019) Learnings about the complexity of extracellular tau aid development of a blood-based screen for Alzheimer's disease. Alzheimers Dement 15:487–496. 30419228 10.1016/j.jalz.2018.09.010 PMC6476313

[B12] Cowan C, Chee F, Shepherd D, Mudher A (2010) Disruption of neuronal function by soluble hyperphosphorylated tau in a *Drosophila* model of tauopathy. Biochem Soc Trans 38:564–570. 10.1042/BST0380564 20298222

[B13] Debanne D, Boudkkazi S, Campanac E, Cudmore R, Giraud P, Fronzaroli-Molinieres L, Carlier E, Caillard O (2008) Paired-recordings from synaptically coupled cortical and hippocampal neurons in acute and cultured brain slices. Nat Protoc 3:1559–1568. 10.1038/nprot.2008.147 18802437

[B14] Fá M, Puzzo D, Piacentini R, Staniszewski A, Zhang H, Baltrons M, Li Puma D, Chatterjee I, Li J, Saeed F, Berman H, Ripoli C, Gulisano W, Gonzalez J, Tian H, Costa J, Lopez P, Davidowitz E, Yu W, Haroutunian V, et al. (2016) Extracellular tao oligomers produce an immediate impairment of LTP and memory. Sci Rep 6 10.1038/srep19393 PMC4726138 26786552

[B15] Fang Q, Hu W, Yang Z (2014) Enhancement of GABA-activated currents by arginine vasopressin in rat dorsal root ganglions. Acta Physiologica Sinica 66:647–657. 25516513

[B16] Harrison PM, Badel L, Wall MJ, Richardson MJE (2015) Experimentally verified parameter sets for modelling heterogeneous neocortical pyramidal cell populations. PLoS Comput Biol 11:e1004165 10.1371/journal.pcbi.1004165 26291316 PMC4546387

[B17] Hoover B, Reed M, Su J, Penrod R, Kotilinek L, Grant M, Pitstick R, Carlson G, Lanier L, Yuan L, Ashe K, Liao D (2010) Tau mislocalization to dendritic spines mediates synaptic dysfunction independently of neurodegeneration. Neuron 68:1067–1081. 10.1016/j.neuron.2010.11.030 21172610 PMC3026458

[B18] Karikari TK, Nagel DA, Grainger A, Clarke-Bland C, Hill EJ, Moffat KG (2019) Preparation of stable tau oligomers for cellular and biochemical studies. Anal Biochem 566:67–74. 10.1016/j.ab.2018.10.013 30315761 PMC6331036

[B19] Karikari T, Turner A, Stass R, Lee L, Wilson B, Nagel D, Hill E, Moffat K (2017) Expression and purification of tau protein and its frontotemporal dementia variants using a cleavable histidine tag. Protein Expr Purif 130:44–54. 10.1016/j.pep.2016.09.009 27663563 PMC5147519

[B20] Kaufmann T, Harrison P, Richardson M, Pinheiro T, Wall M (2016) Intracellular soluble α-synuclein oligomers reduce pyramidal cell excitability. J Physiol 594:2751–2772. 10.1113/JP271968 26915902 PMC4865569

[B21] Kerr M, Wall M, Richardson M (2013) Adenosine A1 receptor activation mediates the developmental shift at layer 5 pyramidal cell synapses and is a determinant of mature synaptic strength. J Physiol 591:3371–3380. 10.1113/jphysiol.2012.244392 23613526 PMC3717233

[B22] Kumar S, Tepper K, Kaniyappan S, Biernat J, Wegmann S, Mandelkow E, Müller D, Mandelkow E (2014) Stages and conformations of the tau repeat domain during aggregation and its effect on neuronal toxicity. J Biol Chem 289:20318–20332. 10.1074/jbc.M114.554725 24825901 PMC4106345

[B23] Lasagna-Reeves C, Castillo-Carranza D, Sengupta U, Guerrero-Munoz M, Kiritoshi T, Neugebauer V, Jackson G, Kayed R (2012) Alzheimer brain-derived tau oligomers propagate pathology from endogenous tau. Sci Rep 2 10.1038/srep00700 PMC3463004 23050084

[B24] Lee V, Goedert M, Trojanowski J (2001) Neurodegenerative tauopathies. Annu Rev Neurosci 24:1121–1159. 10.1146/annurev.neuro.24.1.1121 11520930

[B25] Markram H, Lübke J, Frotscher M, Roth A, Sakmann B (1997) Physiology and anatomy of synaptic connections between thick tufted pyramidal neurones in the developing neocortex. J Physiol 500:409–440. 10.1113/jphysiol.1997.sp022031 9147328 PMC1159394

[B26] McInnes J, Wierda K, Snellinx A, Bounti L, Wang Y, Stancu I, Apóstolo N, Gevaert K, Dewachter I, Spires-Jones T, De Strooper B, De Wit J, Zhou L, Verstreken P (2018) Synaptogyrin-3 mediates presynaptic dysfunction induced by tau. Neuron 97:823–835.e8. 10.1016/j.neuron.2018.01.022 29398363

[B27] Michel C, Kumar S, Pinotsi D, Tunnacliffe A, St. George-Hyslop P, Mandelkow E, Mandelkow E, Kaminski C, Kaminski Schierle G (2014) Extracellular monomeric tau protein is sufficient to initiate the spread of tau protein pathology. J Biol Chem 289:956–967. 10.1074/jbc.M113.515445 24235150 PMC3887218

[B28] Mirbaha H, Chen D, Morazova O, Ruff K, Sharma A, Liu X, Goodarzi M, Pappu R, Colby D, Mirzaei H, Joachimiak L, Diamond M (2018) Inert and seed-competent tau monomers suggest structural origins of aggregation. Elife 7 10.7554/eLife.36584 PMC6039173 29988016

[B29] Mondragón-Rodríguez S, Salas-Gallardo A, González-Pereyra P, Macías M, Ordaz B, Peña-Ortega F, Aguilar-Vázquez A, Orta-Salazar E, Díaz-Cintra S, Perry G, Williams S (2018) Phosphorylation of tau protein correlates with changes in hippocampal theta oscillations and reduces hippocampal excitability in Alzheimer’s model. J Biol Chem 293:8462–8472. 10.1074/jbc.RA117.001187 29632073 PMC5986208

[B30] Moreno H, Morfini G, Buitrago L, Ujlaki G, Choi S, Yu E, Moreira JE, Avila J, Brady ST, Pant H, Sugimori M, Llinás RR (2016) Tau pathology-mediated presynaptic dysfunction. Neuroscience 325:30–38. 10.1016/j.neuroscience.2016.03.044 27012611 PMC4887082

[B31] Nelson PT, Braak H, Markesbery WR (2009) Neuropathology and cognitive impairment in Alzheimer disease: a complex but coherent relationship. J Neuropathol Exp Neurol 68:1–14. 10.1097/NEN.0b013e3181919a48 19104448 PMC2692822

[B32] Ondrejcak T, Klyubin I, Corbett G, Fraser G, Hong W, Mably A, Gardener M, Hammersley J, Perkinton M, Billinton A, Walsh D, Rowan M (2018) Cellular prion protein mediates the disruption of hippocampal synaptic plasticity by soluble tau in vivo. J Neurosci 38:10595–10606. 10.1523/JNEUROSCI.1700-18.2018 30355631 PMC6290298

[B33] Puzzo D, Piacentini R, Fá M, Gulisano W, Li Puma D, Staniszewski A, Zhang H, Tropea M, Cocco S, Palmeri A, Fraser P, D'Adamio L, Grassi C, Arancio O (2017) LTP and memory impairment caused by extracellular Aβ and Tau oligomers is APP-dependent. Elife 6 10.7554/eLife.26991 PMC5529106 28696204

[B34] Qiang L, Sun X, Austin T, Muralidharan H, Jean D, Liu M, Yu W, Baas P (2018) Tau does not stabilize axonal microtubules but rather enables them to have long labile domains. Curr Biol 28:2181–2189.e4. 30008334 10.1016/j.cub.2018.05.045

[B35] Regan P, Piers T, Yi JH, Kim DH, Huh S, Park SJ, Ryu JH, Whitcomb DJ, Cho K (2015) Tau phosphorylation at serine 396 residue is required for hippocampal LTD. J Neurosci 35:4804–4812. 10.1523/JNEUROSCI.2842-14.2015 25810511 PMC4389589

[B36] Reyes A, Sakmann B (1999) Developmental switch in the short term modification of unitary EPSPs evoked in layer 2/3 and layer 5 pyramidal neurons in rat neocortex. J Neurosci 19:3827–3835. 10.1523/JNEUROSCI.19-10-03827.1999 10234015 PMC6782723

[B37] Richardson MJE, Silberberg G (2008) Measurement and analysis of postsynaptic potentials using a novel voltage-deconvolution method. J Neurophysiol 99:1020–1031. 10.1152/jn.00942.2007 18046003

[B38] Rocher AB, Crimins JL, Amatrudo JM, Kinson MS, Todd-Brown MA, Lewis J, Luebke JI (2010) Structural and functional changes in tau mutant mice neurons are not linked to the presence of NFTs. Exp Neurol 223:385–393. 10.1016/j.expneurol.2009.07.029 19665462 PMC2864360

[B39] Rudinskiy N, Hawkes J, Wegmann S, Kuchibhotla K, Muzikansky A, Betensky R, Spires-Jones T, Hyman B (2014) Tau pathology does not affect experience-driven single-neuron and network-wide Arc/Arg3.1 responses. Acta Neuropathol Commun 2:63 10.1186/2051-5960-2-63 24915991 PMC4229905

[B40] Shammas S, Garcia G, Kumar S, Kjaergaard M, Horrocks M, Shivji N, Mandelkow E, Knowles T, Mandelkow E, Klenerman D (2015) A mechanistic model of tau amyloid aggregation based on direct observation of oligomers. Nat Commun 6:7025 10.1038/ncomms8025 25926130 PMC4421837

[B41] Spires T, Orne J, SantaCruz K, Pitstick R, Carlson G, Ashe K, Hyman B (2006) Region-specific dissociation of neuronal loss and neurofibrillary pathology in a mouse model of tauopathy. Am J Pathol 168:1598–1607. 10.2353/ajpath.2006.050840 16651626 PMC1606598

[B42] Tamagnini F, Walsh DA, Brown JT, Bondulich MK, Hanger DP, Randall AD (2017) Hippocampal neurophysiology is modified by a disease-associated C-terminal fragment of tau protein. Neurobiol Aging 60:44–56. 10.1016/j.neurobiolaging.2017.07.005 28917666 PMC5654728

[B43] Tanemura K, Murayama M, Akagi T, Hashikawa T, Tominaga T, Ichikawa M, Yamaguchi H, Takashima A (2002) Neurodegeneration with tau accumulation in a transgenic mouse expressing V337M human tau. J Neurosci 22:133–141. 11756496 10.1523/JNEUROSCI.22-01-00133.2002 PMC6757582

[B44] Tatebayashi Y, Miyasaka T, Chui D, Akagi T, Mishima K, Iwasaki K, Fujiwara M, Tanemura K, Murayama M, Ishiguro K, Planel E, Sato S, Hashikawa T, Takashima A (2002) Tau filament formation and associative memory deficit in aged mice expressing mutant (R406W) human tau. Proc Natl Acad Sci USA 99:13896–13901. 10.1073/pnas.202205599 12368474 PMC129794

[B45] Teravskis P, Covelo A, Miller E, Singh B, Martell-Martínez H, Benneyworth M, Gallardo C, Oxnard B, Araque A, Lee M, Liao D (2018) A53T mutant alpha-synuclein induces tau-dependent postsynaptic impairment independently of neurodegenerative changes. J Neurosci 38:9754–9767. 10.1523/JNEUROSCI.0344-18.2018 30249789 PMC6222065

[B46] Tracy T, Gan L (2018) Tau-mediated synaptic and neuronal dysfunction in neurodegenerative disease. Curr Opin Neurobiol 51:134–138. 10.1016/j.conb.2018.04.027 29753269 PMC6130905

[B47] Uhlenbeck GE, Ornstein LS (1930) On the theory of the Brownian motion. Phys Rev 36:823–841. 10.1103/PhysRev.36.823

[B48] von Bergen M, Barghorn S, Biernat J, Mandelkow E-M, Mandelkow E (2005) Tau aggregation is driven by a transition from random coil to beta sheet structure. Biochim Biophys Acta 1739:158–166. 10.1016/j.bbadis.2004.09.010 15615635

[B49] Wittman CW, Wszolek MF, Shulman JM, Salvaterra PM, Lewis J, Hutton M, Feany MB (2001) Tauopathy in *Drosophila*: neurodegeneration without neurofibrillary tangles. Science 293:711–714. 11408621 10.1126/science.1062382

[B50] Yoshiyama Y, Higuchi M, Zhang B, Huang S, Iwata N, Saido T, Maeda J, Suhara T, Trojanowski J, Lee V (2007) Synapse loss and microglial activation precede tangles in a P301S tauopathy mouse model. Neuron 53:337–351. 10.1016/j.neuron.2007.01.010 17270732

[B51] Zhou L, McInnes J, Wierda K, Holt M, Herrmann AG, Jackson RJ, Wang YC, Swerts J, Beyens J, Miskiewicz K, Vilain S, Dewachter I, Moechars D, De Strooper B, Spires-Jones TL, De Wit J, Verstreken P (2017) Tau association with synaptic vesicles causes presynaptic dysfunction. Nat Commun 8:15295. 28492240 10.1038/ncomms15295 PMC5437271

