# Supplementary material for: Introduction of Tau Oligomers into Cortical Neurons Alters Action Potential Dynamics and Disrupts Synaptic Transmission and Plasticity
Source: eNeuro. 2019 Oct 14;6(5):ENEURO.0166-19.2019. doi: 10.1523/ENEURO.0166-19.2019 (PMC6794083; doi:10.1523/ENEURO.0166-19.2019)
Supplement: Extended Data Table 1-1 — Electrophysiological parameters measured for single layer V cells, during paired layer V recordings and for CA1 neurons in the long-term potentiation experiments. Data is shown as mean, standard error of the mean (SEM) and standard deviation (SD). Download Table 1-1, DOC file. [file sup_enu-eN-NWR-0166-19-s01.doc]

**Table 1-1**

| **Condition** |  | **Time** | **Mean** | **SEM** | **SD** |
| --- | --- | --- | --- | --- | --- |
|  | **Single cell recording - Layer V** |  |  |  |  |
| 666 nM oTau | Action potential amplitude - reduction (mV) | *0 mins* | 74.45 | 2.13 | 8.52 |
|  |  | *40 mins* | 60.135 | 6.38 | 25.52 |
| Vehicle | Action potential amplitude - no change (mV) | *0 mins* | 80.85 | 1.82 | 9.1 |
|  |  | *40 mins* | 77.87 | 2.08 | 10.4 |
| 666 nM oTau | Input resistance - increase (MΩ) | *0 mins* | 92.5 | 7.58 | 30.32 |
|  |  | *40 mins* | 108.19 | 8.107 | 32.428 |
| Vehicle | Input resistance - no change (MΩ) | *0 mins* | 90.1 | 11.95 | 59.75 |
|  |  | *40 mins* | 92.15 | 12.99 | 64.95 |
| 666 nM oTau | No change to resting membrane potential (mV) | *0 mins* | 67.5 | 1.5 | 6 |
|  |  | *40 mins* | 62.5 | 1.3 | 5.2 |
|  | **Paired recordings - Layer V** |  |  |  |  |
| Vehicle | Mean EPSP amplitude (mV) | *NA* | 0.75 | 0.13 | 0.65 |
| 666 nM oTau | Mean EPSP amplitude (mV) | *NA* | 0.16 | 0.13 | 0.65 |
| Vehicle | Mean EPSP latency (ms) | *NA* | 2.2 | 0.0002 | 0.001 |
| 666 nM oTau | Mean EPSP latency (ms) | *NA* | 2.1 | 0.0001 | 0.0005 |
|  |  |  |  |  |  |
|  | **Synaptic Plasticity - Long Term potentiation** |  |  |  |  |
| Vehicle | Mean potentiation (% baseline) | *NA* | 3.31 | 1.32 | 4.62 |
| L689,560 | Mean potentiation (% baseline) | *NA* | 0.53 | 0.14 | 0.21 |
| 444 oTau | Mean potentiation (% baseline) | *NA* | 0.989 | 0.31 | 1.085 |
| 44 oTau | Mean potentiation (% baseline) | *NA* | 1.11 | 0.1 | 0.25 |
| 444 mTau | Mean potentiation (% baseline) | *NA* | 3.35 | 0.9 | 2.25 |

Table 1-1: Electrophysiological parameters measured for single layer V cells, during paired layer V recordings and for CA1 neurons in the long-term potentiation experiments. Data is shown as mean, standard error of the mean (SEM) and standard deviation (SD).
